# Transient lung eosinophilia during breakthrough influenza infection in vaccinated mice is associated with protective and balanced Type 1/2 immune responses

**DOI:** 10.1128/jvi.00965-25

**Published:** 2025-11-05

**Authors:** Lauren A. Chang, Stephen T. Yeung, Prajakta Warang, Moataz Noureddine, Gagandeep Singh, Brett T. Webb, Eleanor Burgess, Michael Schotsaert

**Affiliations:** 1 Department of Microbiology, Icahn School of Medicine at Mount Sinai 200769, New York, New York, USA; 2 Graduate School of Biomedical Sciences, Icahn School of Medicine at Mount Sinai 5925, New York, New York, USA; 3 Global Health and Emerging Pathogens Institute, Icahn School of Medicine at Mount Sinai 5925, New York, New York, USA; 4 Department of Medicine, Division of Infectious Diseases, Weill Cornell Medical College 12295 , New York, New York, USA; 5 Veterinary Diagnostic Laboratory, North Dakota State University 3323, Fargo, North Dakota, USA; 6 Lipschultz Precision Immunology Institute (PrIISM), Icahn School of Medicine at Mount Sinai 5925, New York, New York, USA; 7 Icahn Genomics Institute, Icahn School of Medicine at Mount Sinai 5925, New York, New York, USA; University Medical Center Freiburg, Freiburg, Germany

**Keywords:** innate immunity, breakthrough infection, eosinophil, influenza, influenza vaccine

## Abstract

Eosinophils are versatile cells that participate in a multitude of homeostatic and inflammatory responses in the lung, ranging from allergic asthma to antiviral defense against respiratory viral infection. In the context of vaccination followed by viral infection, such as breakthrough infection, eosinophils have been linked to aberrant Th2 responses like vaccine-enhanced respiratory disease. Here, we demonstrate that the lung immune cell composition, cytokine and chemokine repertoire, histopathological profile, and systemic humoral response of breakthrough influenza infection in mice are distinct from those of primary influenza infection or allergic sensitization, canonical Type 1 and 2 immune responses, respectively. Longitudinal comparison of breakthrough infection with allergic sensitization and primary influenza infection demonstrated major differences in lung immunity between treatment groups in female BALB/c mice. Breakthrough infection mice exhibit lung eosinophil infiltration that peaks at 7–10 days post-challenge, enriched for the Siglec-F^hi^ subset, but in the absence of overt pro-inflammatory cytokine/chemokine signals, high viral titers, severe lung lesions, goblet cell hyperplasia, allergic levels of total IgE, or enhanced morbidity. Multiparameter fluorescence imaging corroborated findings from flow cytometry and also unveiled interactions between CD101^+^Siglec-F^+^ cells and CD3^+^ cells in the lung tissue space. Imaging also revealed a marked absence of eosinophil or neutrophil extracellular traps in the lungs of breakthrough infection mice, in contrast with allergic sensitization and primary influenza infection, respectively. Altogether, our findings provide a deeper understanding of the kinetics and cell-cell interplay during non-pathological, balanced Type 1/2 immune responses in vaccinated hosts during breakthrough infection.

**IMPORTANCE:**

Our findings reveal that eosinophilic recruitment during influenza breakthrough infection is non-pathological and represents a balanced immune response, distinct from highly inflammatory environments seen in primary influenza infection or allergic sensitization. We observed eosinophil enrichment in the lungs of vaccinated hosts following infection, which coincided with rapid viral clearance and minimal lung damage, challenging traditional associations of eosinophils with adverse outcomes in vaccinated hosts, like for respiratory syncytial virus. We identified a phenotypic shift toward Siglec-F^hi^ eosinophil subset in breakthrough infection mice. Additionally, we highlight preservation of alveolar macrophages and absence of neutrophil and eosinophil extracellular traps in vaccinated hosts, key features that distinguish breakthrough infection from both Type 1- and Type 2-skewed disease models. These insights have broad implications for exploring eosinophil plasticity and cell-cell interactions in the lung, which could inform the development of strategies to harness the benefits of balanced Type 1/2 immune responses across different vaccine/respiratory virus pairs.

## INTRODUCTION

Humans are repeatedly infected with influenza over the course of their lifetime. Although seasonal vaccination is able to reduce the incidence of transmission and severe illness, current clinically approved vaccines have variable efficacy (1–4). Breakthrough infections of vaccinated individuals can still occur, but with substantially lower disease severity (5). This is partially conferred by immune cells like memory CD8 T cells, generated by previous vaccination or infection, which can kill infected cells by recognizing conserved epitopes presented in the context of major histocompatibility complex (MHC) proteins (6). Innate immune cells can also aid in control of the virus during breakthrough infection, extrapolating from their roles during primary influenza infection (7). For example, neutrophils, monocytes, and alveolar macrophages (AMs) can assist by phagocytic clearance of infected dead cells from virus-infected lungs (7, 8). Another innate immune cell, the eosinophil, also has many documented functions during primary influenza infection (9–12). Eosinophils can exert both direct antiviral functions, such as killing via release of granule proteins, or indirect functions such as priming T cells as non-professional antigen-presenting cells (13). Eosinophils are also involved in the tissue repair response, such as after virus-induced damage (14, 15).

Despite the many antiviral capabilities of eosinophils, the appearance of these cells during respiratory viral breakthrough infection of vaccinated hosts has a negative connotation. This is in part due to the association of eosinophils with vaccine-enhanced respiratory disease (VAERD) following alum-adjuvanted formalin-inactivated respiratory syncytial virus vaccine (FI-RSV) trials in infants during the 1960s, where vaccinated children had severe, clinical disease following natural RSV infection, including more frequent hospitalization (16, 17). VAERD is characterized by elevated levels of canonical Type 2 cytokines (IL-4, IL-5, IL-13), elevated eosinophil counts, and increased pathology in vaccinated populations after encountering wild-type virus relative to unvaccinated populations (17). All of these hallmarks, alongside increased mucin production and goblet cell hyperplasia, have been described in mouse models for RSV VAERD (18). However, a thorough investigation has pinpointed Th2-biased CD4 T-cell responses and generation of low-avidity non-neutralizing antibodies as major drivers of RSV VAERD etiology, and not eosinophils (17–19). Influenza VAERD has been demonstrated in pigs, although the likely cause appears to be elicitation of non-neutralizing antibodies rather than aberrant Th2-skewing of CD4 T cells (20–22).

Our lab has optimized a breakthrough influenza infection model and has observed a dose-dependent recruitment of eosinophils to the lungs following challenge; mice that received three vaccinations had more lung eosinophils at 7 days post-challenge than mice that received one dose (23). Eosinophils did not appear to correlate with the severity of lesions, but rather with enhanced protection in stark contrast with VAERD. We further characterized this phenotype as a non-pathological enrichment of lung eosinophils, particularly that of the more inflammatory phenotype (Siglec-F^hi^ CD101^+^), in the absence of aberrant Type 1 or Type 2 inflammation, high concentrations of total serum IgE, or morbidity during influenza breakthrough infection (24, 25). Despite the enrichment of eosinophils in the lung at 7 days post-challenge (DPC), no significant IL-5 or Eotaxin-1 (CCL11) signature was observed in lung homogenate supernatant cytokine/chemokine analysis, in contrast to observations in OVA-sensitized, allergic, positive control mice with robust eosinophilia, which exhibited high concentrations of IL-5 and CCL11 (24). Furthermore, the ratio of IgG2a/IgG1 as a surrogate of Th1/Th2 skewing did not track with the degree of eosinophil infiltration in breakthrough infection mice, since mice exhibiting the highest degree of lung eosinophilia had balanced IgG2a/IgG1 ratios rather than strongly IgG1-skewed ratios that would be indicative of Th2-biased immunity. Adjuvanting the vaccine with a strong Th1-skewing agent also did not deter eosinophil influx. Lung eosinophilia after breakthrough infection at 7 DPC was also observed in male BALB/c mice, indicating sex independence. A vaccine-mismatched challenge in mice also recruited eosinophils to the lung. Collectively, our previous findings suggested that peripheral priming of hosts via intramuscular vaccination followed by a viral exposure in the lung resulted in non-allergic, non-pathogenic, complete resolution of respiratory viral disease, coinciding with eosinophilia rather than VAERD. The exact function of the recruited lung eosinophil subsets during breakthrough infection remains to be determined.

Given that previous studies by Choi et al. (23) and Chang et al. (24) assessed lung immunity at a singular time point (7 DPC), we conducted a longitudinal study with both the OVA allergic sensitization model and the influenza breakthrough infection model to better compare and contrast the kinetics of both immune responses. Primary influenza infection was also included as a condition to allow for direct comparison of breakthrough infection with a typical Type 1 immune reaction, alongside OVA allergic sensitization as a positive control for a canonical Type 2 response. After determining the time point where peak eosinophilia is observed, especially that of the Siglec-F^hi^ subset, we conducted multicolor fluorescence imaging. We visually confirmed eosinophilia in the lungs at 7 DPC and observed granulocyte-T-cell interactions as well. Furthermore, we saw neutrophil and eosinophil extracellular trap formation in the primary influenza infection and OVA-sensitized mice, respectively, but not in the breakthrough infection mice. Therefore, eosinophil influx during influenza infection in the context of pre-existing immunity (breakthrough) may reflect different functionality compared to allergy-induced eosinophilia.

## MATERIALS AND METHODS

### Study design

Female, 6- to 8-week-old BALB/c mice were obtained from The Jackson Laboratory (Bar Harbor, ME). Mice were housed under specific pathogen-free conditions with food and water provided *ad libitum*. All experiments were approved by and performed according to the Icahn School of Medicine at Mount Sinai Institutional Animal Care and Use Committee.

The vaccine used in this study was obtained through BEI Resources, NIAID, NIH: Fluzone Influenza Virus Vaccine, 2005–2006 Formula, NR-10480. Mice were vaccinated intramuscularly in the hind limbs with a seasonal trivalent inactivated influenza virus vaccine (TIV; Fluzone Influenza Virus Vaccine) containing an influenza A H1N1 component (A/New Caledonia/20/1999/IVR-116), influenza A H3N2 component (A/New York/55/2004/X-157 [an A/California/7/2004-like strain]), and influenza B component (B/Jiangsu/10/2003 [a B/Shanghai/361/2002-like strain]). Phosphate-buffered saline (PBS) was injected as a negative control for vaccination. Non-adjuvanted TIV or PBS was administered intramuscularly to both hind limbs (50 µL/limb, 100 µL/mouse total). Five mice were used per treatment group per time point.

### Influenza challenge

Mice were challenged with a sublethal (0.2 LD_50_) dose of H1N1 A/New Caledonia/20/1999 (NC99), as described previously for the breakthrough infection model (23, 24). Briefly, mice were administered a mixture of ketamine and xylazine intraperitoneally (i.p.) for anesthesia. Mice then received a 50 µL intranasal (i.n.) challenge of either mouse-adapted egg-grown influenza virus or egg allantoic fluid as a vehicle control. Body weights were recorded immediately before the challenge to define 100% body weight, then mice were weighed daily through 10 days post-infection to monitor morbidity. Additional body weight measurements were recorded for relevant groups at 28–30 days post-challenge prior to harvest.

### OVA sensitization

Mice were sensitized to OVA as described previously (24). Briefly, mice were sensitized twice with a 100 µL i.p. injection containing 20 µg of Imject ovalbumin (OVA) (Thermo Scientific, cat. no. 77120) adsorbed to alum, administered 1 week apart. Negative control mice received a 100 µL i.p. injection of PBS. One week after the final sensitization, mice were anesthetized as described above and i.n. challenged with 20 µg OVA diluted in PBS (final volume of 50 µL/mouse). Control mice did not receive an intranasal challenge. Three mice were used per treatment group at each time point. Body weights were recorded as described above.

### Serum collection

Blood was collected from the submandibular vein and coagulated at 4°C overnight. The next day, coagulated blood was centrifuged at 400 × *g* for 5 minutes at 4°C. Serum was collected and stored at −20°C until analysis.

### Flow cytometry

Mice were euthanized with sodium pentobarbital. The left lung lobe was collected into a C-tube (Miltenyi Biotec, cat. no. 130-096-334) filled with 2.4 mL of 1× Buffer S from the Miltenyi Biotec Mouse Lung Dissociation Kit (cat. no. 130-095-927), diluted as per the manufacturer’s instructions. 100 µL of Enzyme D and 15 µL of Enzyme A were spiked into each C-tube and then digested on a Miltenyi Biotec gentleMACS Octo Dissociator with Heaters using the 37C_m_LDK_1 protocol. Digested lungs were then centrifuged at 400 × *g* for 5 minutes, and the supernatant was discarded. Pellets were resuspended in 5 mL of 1× Red Blood Cell (RBC) Lysis Buffer (8.29 g NH_4_Cl, 1.00 g KHCO_3_, and 200 µL of 0.5 M EDTA dissolved in ddH_2_O) and then incubated at room temperature for 5 minutes, before centrifugation at 400 × *g* for 5 minutes. After RBC lysis, pellets were resuspended with 5 mL of PBS, passed through a 70 µm cell strainer (Greiner, cat. no. 542170), then centrifuged at 400 × *g* for 5 minutes. Supernatants were discarded, and cell pellets were resuspended with 200 µL of Flow Cytometry (FC) Buffer (1% BSA, 2 mM EDTA in 1× PBS) before transferring the cell suspension to a 96-well V-bottom polypropylene plate (Thermo Scientific, cat. no. 249944). Plates were centrifuged at 400 × *g* for 5 minutes, supernatants were discarded, and pellets were stained for flow cytometry as described previously (24). Briefly, pellets were resuspended with 50 µL of Purified Rat Anti-Mouse CD16/CD32 Fc Block (clone 2.4G2, BD) diluted 1:100 in FC Buffer and incubated at room temperature for 5 minutes in the dark. Subsequently, 50 µL of surface staining antibody cocktail was added to each well of Fc-blocked cells, bringing the total well volume to 100 µL. The cocktail contained the following antibodies and dyes: CD11c FITC (1:150, clone HL3, BD), CD125 PE (1:150, clone T21, BD), Siglec-F PE-CF594 (1:150, clone E50-2440, BD), Ly6G PerCP-Cy5.5 (1:150, clone 1A8, BD), CD101 PE-Cy7 (1:150, clone Moushi101, Invitrogen), CD11b APC (1:150, clone M1/70, BioLegend), MHC II Alexa Fluor 700 (1:150, clone M5/114.15.2, Invitrogen), CD62L APC-Cy7 (1:150, clone MEL-14, BD), and Fixable Viability Dye eFluor 450 (1:200, eBioscience). Cells were incubated with the surface staining cocktail at room temperature for 20 minutes in the dark. To wash, 120 µL of FC Buffer was added on top of the cells, followed by centrifugation at 400 × *g* for 5 minutes. Supernatants were decanted, plates were blotted on paper towels, then a second wash was performed by resuspending pellets with 200 µL of FC Buffer followed by centrifugation at 400 × *g* for 5 minutes. After washing, cell pellets were resuspended in 200 µL of FC Buffer, and 5 µL of CountBright Absolute Counting Beads (ThermoFisher) was added to all samples. For single-stained compensation controls, 1 µl of antibody was added to 1 drop of UltraComp eBeads Plus Compensation Beads (ThermoFisher) for each antibody in the panel. Samples were acquired on a Beckman Coulter Gallios flow cytometer with Kaluza software. Data were analyzed using FlowJo v10 (BD), and raw data were compensated using AutoSpill. Following manual gating in FlowJo, data were visualized and statistically analyzed in GraphPad Prism version 10.2.1.

### Determination of infectious viral titer

Lung middle, inferior, and post-caval lobes were collected in 500 µL of sterile PBS in prefilled homogenizer bead tubes containing 3.0 mm high-impact zirconium beads (Benchmark Scientific, cat. no. D1032-30). Samples were snap-frozen on dry ice on the day of harvest immediately after collection, then stored at −80°C until analysis. Samples were thawed on ice on the day of the assay, then homogenized and centrifuged at 10,000 × *g* for 5 minutes at 4°C to clarify lung homogenate supernatants.

To determine the 50% tissue culture infectious dose (TCID_50_), 2 × 10^4^ Madin-Darby canine kidney cells (ATCC, cat. no. CCL-34) were seeded per well at a final volume of 100 µL in Dulbecco’s Modified Eagle Medium (DMEM) (Gibco) containing 10% heat-inactivated fetal bovine serum (FBS) and 100 U/mL Penicillin-Streptomycin (Gibco) in a 96-well flat-bottom plate (Corning), then incubated at 37°C for 24 h. On the day of the assay, clarified lung homogenate supernatants were serially diluted 1:10 for a total of 8 dilutions in 96-well U-bottom plates (Corning) in serum-free DMEM containing 100 U/mL Penicillin-Streptomycin and 1 µg/mL TPCK trypsin: 8 µL of sample was diluted into 72 µL media for each dilution. After all samples were diluted, the serum-containing medium was removed from the cell plates, and the cell plates were then washed twice with 200 µL of sterile PBS per well and then patted dry on paper towels. Immediately after washing, 50 µL of diluted sample was transferred from the dilution plates to the cell plates and incubated at 37°C for 1 h. After the incubation, 100 µL of serum-free media was added to each well of the cell plates. Plates were then incubated at 37°C for 72 h. After incubation, supernatants were discarded, and the cells were fixed with 200 µL of 5% formaldehyde in PBS per well and incubated at 4°C overnight. The next day, supernatants were discarded and plates were washed 3 times with 200 µL/well of PBS-T (1× PBS with 0.05% Tween-20). Plates were then blocked with 200 µL/well of 5% milk in PBS-T for 1 h at room temperature. After blocking, plates were washed three times as described, and 100 µL/well of primary antibody solution (hyper-immune mouse serum diluted 1:500 in 5% milk in PBS-T) was added to the plates. After incubating for 1 h at room temperature, plates were washed three times as described. The goat anti-mouse IgG-HRP detection antibody (Abcam, cat. no. ab6823) was diluted 1:5,000 in 5% milk in PBS-T, then 100 µL/well was added to the washed plates. After incubating for 1 h at room temperature, plates were washed three times as described. Plates were then washed one additional time with 1× PBS. After washing, 100 µL/well of 1-Step Turbo TMB Substrate (ThermoFisher, cat. no. 34022) was added to the plates and incubated for 20 minutes at room temperature in the dark. After the incubation, the reaction was quenched by adding 100 µL/well of enzyme-linked immunosorbent assay (ELISA) Stop Solution (Invitrogen, cat. no. SS04). Plates were read on a BioTek Synergy Neo2 (Agilent) plate reader at 450 nm and 650 nm. Background-subtracted optical density values (OD_450-650nm_) were used for data analyses. The TCID_50_ was determined using the Reed-Muench method (26). Positive wells were defined as wells containing an OD_450-650nm_ value >2× the average of the OD_450–650 nm_ values of the media-only wells.

### Bead-based multiplex cytokine/chemokine assay

Lung lobes were collected and processed into clarified lung homogenate supernatants as described above for the TCID_50_ assay. Bead-based multiplex cytokine/chemokine assays were performed on the same day as the TCID_50_ assay to minimize sample freeze-thaw number. To measure a total of 27 lung cytokines and chemokines, we used the ProcartaPlex Mouse Cytokine & Chemokine Panel 1, 26plex kit (ThermoFisher, cat. no. EPX260-26088-901,) in conjunction with the IL-33 Mouse ProcartaPlex Simplex Kit (ThermoFisher, cat. no. EPX010-26025-901). Kits were combined, and the assay was performed as described by the manufacturer. For all incubation steps, unless stated otherwise, plates were placed on an orbital shaker set to 300 rpm. Briefly, 100 µL/well of clarified lung homogenate supernatant was added to washed beads in a black, optical flat-bottom 96-well plate and incubated for 30 minutes at room temperature protected from light. After the room temperature incubation, plates were placed on a flat surface at 4°C for overnight incubation. The following day, the plate was incubated for 30 minutes at room temperature, then washed 3 times with 150 µL/well of 1× Wash buffer, prepared according to the kit instructions. After washing, the 1× Detection Antibody cocktail was combined as recommended by the manufacturer, and 25 µL/well was added. Plates were incubated at room temperature for 30 minutes, protected from light. After incubation, plates were washed three times as described and 50 µL/well of 1× Streptavidin-PE solution was added to the plates. Plates were incubated for 30 minutes at room temperature, protected from light. Plates were then washed three times as described, and 120 µL/well of Reading Buffer was added. Plates were incubated for 5 minutes at room temperature and acquired on a Luminex 100/200 analyzer (Millipore) with xPONENT software (version 4.3). Extrapolated data were exported from the xPONENT software and visualized in GraphPad Prism version 10.2.1 or with *pheatmap* using the R computing language (ver. 4.05) in RStudio (ver. 1.4.1106).

### Tissues and histochemical staining

The right superior lobes were collected in 10% buffered formalin. Tissues were processed whole, embedded, sectioned at 4–5 microns, and stained with hematoxylin and eosin (H&E) by routine methods and scored as described in Table 1. Alcian blue periodic acid Schiff (AB-PAS) stains were performed as previously described on 4–5 micron sections (27).

**TABLE 1:** Scoring criterion for histopathological findings

Score	Area affected	Epithelial degeneration/necrosis	Inflammation	Atelectasis
0	None	None	None	None
1	<10%	Minimal; scattered rare cells affecting <10% of the tissue section	Rare scattered cells affecting <10% of the tissue section	Minimal
2	10%–25%	Mild; scattered cell necrosis/vacuolation affecting 10%–25% of thetissue section	Scattered inflammatory cells affecting 10%–25% of the tissue section	Mild
3	25%–50%	Moderate; multifocal vacuolation or sloughed/necrotic cells	Thin layer of cells (<5 cell layers thick)	Moderate
4	50%–75%	Marked; multifocal/segmental necrosis, epithelial loss/effacement	Thick layer of cells (>5 cell layers thick)	Marked
5	>75%	Severe; coalescing areas of necrosis, parenchymal effacement	Confluent areas of inflammation	Severe

### Histopathology

On H&E-stained sections, the percent area affected, epithelial degeneration and necrosis, inflammation, and atelectasis were graded on a 0–5+ scale as detailed in Table 1. AB-PAS-stained sections were graded on a 1–5+ scale based on the number of positively staining cells in airways using the following scale: 1+ = no positively cells, 2+ = occasional positively staining cells, 3+ = low numbers of positively staining cells, 4+ = moderate numbers of positively staining cells, and 5+ = nearly confluent positively staining cells. Staining was categorized based on predominant cytoplasmic staining among the cells: AB positive, PAS positive, or mixed, and presence or absence of a main bronchus within the section. One mouse was omitted from analyses of pathological data due to the presence of background pneumonia in the lobe, unrelated to treatment, as per the veterinary pathologist’s recommendation.

### Enzyme-linked immunosorbent assays

To measure OVA-specific antibody titers in the OVA-sensitized mice, Nunc MaxiSorp 96-well plates (ThermoFisher, cat. no. 456537) were coated with 100 µL/well of Imject OVA diluted to a concentration of 10 µg/mL in carbonate-bicarbonate buffer and incubated overnight at 4°C. The next day, plates were washed three times with 200 µL/well of PBS-T (1X PBS with 0.05% Tween-20) and patted dry on paper towels. Plates were then blocked with 200 µL/well of blocking buffer (5% milk in PBS-T). While plates were blocking for 1 h at room temperature, sera were serially diluted in blocking buffer fourfold, a total of seven times, beginning from a 1:25 dilution (total IgG) or a 1:50 dilution (IgG1, IgG2a). Serial dilutions were performed in a 96-well V-bottom polypropylene plate (Thermo Scientific, cat. no. 249944). Plates were washed three times as described after blocking, then 50 µL/well of diluted sera was added to the plates, incubating for 1 h at room temperature. After incubation with serially diluted sera, plates were washed three times and 100 µL/well of diluted detection antibody was added: goat anti-mouse IgG-HRP (1:5,000, Abcam, cat. no. ab6823), goat anti-mouse IgG1-HRP (1:4,000, SouthernBiotech, cat. no. 1071-05), or goat anti-mouse IgG2a-HRP (1:4,000, SouthernBiotech, cat. no. 1081-05) diluted in blocking buffer. Plates were incubated for 1 h at room temperature. After incubation with diluted detection antibody, plates were washed three times with PBS-T as described, followed by an additional wash with 1× PBS. Plates were then developed by adding 100 µL/well of 1-Step Turbo TMB Substrate (ThermoFisher), incubating for 20 minutes at room temperature in the dark. After incubation, 100 µL/well of ELISA Stop Solution (Invitrogen) was added to quench the reaction.

To measure vaccine-specific antibody titers in the breakthrough infection mice, Nunc MaxiSorp 96-well plates (ThermoFisher) were coated with 50 µL/well of TIV diluted 1:250 in carbonate-bicarbonate buffer and incubated overnight at 4°C. The next day, plates were washed and blocked as described above for 1 h at room temperature. The serum was diluted in blocking buffer fivefold a total of seven times beginning from a 1:150 dilution (total IgG), or threefold a total of seven times beginning from a 1:100 dilution (IgG1, IgG2a). As described above, plates were washed three times after blocking, then 100 µL/well of diluted sera was added to the plates, and then incubated for 1 h at room temperature. Plates were washed three times, then 100 µL/well of diluted detection antibody was added as described above, for each respective isotype, and incubated for 1 h at room temperature. After incubation with diluted detection antibody, plates were washed and developed as described above.

To measure total IgE, Nunc MaxiSorp 96-well plates (ThermoFisher) were coated with 100 µL/well of anti-mouse IgE antibody (Clone R35-72, BD) diluted to a concentration of 2 µg/mL in carbonate-bicarbonate buffer and incubated overnight at 4°C. The next day, plates were washed and blocked as described above for 1 h at room temperature. As plates were blocked, each serum sample was diluted 1:50 in blocking buffer in technical duplicates in a 96-well V-bottom polypropylene plate (Thermo Scientific, cat. no. 249944). Unlabeled, purified mouse IgE (SouthernBiotech, cat. no. 0114-01) was diluted fourfold a total of 11 times from a starting concentration of 4,000 ng/mL to generate a standard curve. After blocking, plates were washed three times as described, and 50 µL/well of diluted sera was added to the plates and incubated for 1 h at room temperature. After incubation with sera, plates were washed and then incubated with 100 µL/well of goat anti-mouse IgE-HRP (SouthernBiotech, cat. no. 1110-05) diluted to 1:4,000 in blocking buffer for 1 h at room temperature. After incubation with diluted detection antibody, plates were washed and developed as described above.

For all ELISAs, plates were read on a BioTek Synergy Neo2 (Agilent) plate reader at 450 nm and 650 nm. Background-subtracted optical density values (OD_450-650nm_) were used for data analyses. Endpoint titers and interpolated total IgE concentrations were calculated in GraphPad Prism version 10.2.1.

### Principal component analysis

The following metrics from each mouse were integrated into a singular data frame for principal component analysis (PCA): flow cytometry absolute numbers (5 populations of interest); body weight percentages (up to 12 timepoints); concentrations of 27 different cytokines and chemokines measured from clarified lung homogenate supernatants; histopathology scores for 19 different metrics, including total score; goblet cell scores; and total IgE concentration (three timepoints) for a total of 67 different metrics. OVA- and vaccine-specific total IgG, as well as TCID_50_ values, were omitted from analyses to facilitate concomitant antigen-agnostic comparison of host immune profiles observed in the OVA sensitization and breakthrough infection studies. Data visualization and analysis were conducted using *prcomp* in the R computing language (ver. 4.05) in RStudio (ver. 1.4.1106).

### Tissue preparation for immunofluorescence and confocal microscopy

Briefly, tissues were fixed in paraformaldehyde, lysine, and sodium periodate buffer (PLP, 0.05 M phosphate buffer, 0.1 M L-lysine, pH 7.4, 2 mg/mL NaIO4, and 10 mg/mL paraformaldehyde) overnight at 4°C, followed by 30% sucrose overnight at 4°C and subsequent embedding in OCT media. 20 µm frozen tissue sections were sectioned using a Leica CM3050S cryostat (Leica Biosystems Inc). FcR was blocked with anti-CD16/32 Fc block antibody (clone 93, BioLegend) diluted in 1× PBS containing 2% donkey serum and 2% FBS for 1 h at room temperature. Sections were stained with Siglec-F BV421 (Clone E50-2440, BD), CD11b AF488 (Clone M1/70, BioLegend), CD11c PE (Clone N418, Invitrogen), Ly6G APC (Clone 1A8, BioLegend), Ly6G BV510 (Clone 1A8, BD), CD62L AF488 (Clone MEL-14, Southern Biotech), CD101 PE (Clone Moushi101, Invitrogen), CD11c eFluor 615 (Clone N418, Invitrogen), CD125 APC (Clone DIH37, BioLegend), CD3e APC (Clone 145-2C11, BioLegend), B220 V500 (Clone RA3-6B2, BD), Ly6G AF488 (Clone 1A8, BioLegend), anti-EPX (Clone MM25.8.2.2, antibody kindly provided by Dr. Elizabeth Jacobsen), or anti-Citrullinated Histone H3 (Abcam) diluted in PBS containing 2% donkey serum, 2% FBS, and 0.5% Fc block for 1 h at room temperature. Sections were subsequently washed with 1× PBS to remove unbound antibody. Following, sections were stained with goat anti-mouse AF488 (Invitrogen) and goat anti-rabbit AF546 (Invitrogen) secondary antibodies diluted in 1× PBS containing 2% donkey serum, 2% FBS, and 0.5% Fc block for 1 h at room temperature. Sections were nuclear stained with DRAQ7 (ThermoFisher, Waltham, MA, USA) for 5 minutes at room temperature and then washed with 1× PBS. Slides were subsequently washed with 1× PBS and cover slipped using Immunmount mounting medium (Fisher Scientific) and Cover Glasses with a 0.13–0.17 mm thickness (Fisher Scientific). Fluorescence was detected with a Zeiss LSM 880 confocal microscope (Carl Zeiss) equipped with 405, 488, 514, 561, 594, and 633 nm solid-state laser lines, a 32-channel spectral detector (409–695 nm), and 10 × 0.3, 20× Plan-Apochromat 0.8, 40×, and 6331.40 objectives. The Zen Black (Carl Zeiss) software suite was used for data collection. The imaging data were processed using Imaris software version 8.3.1 (Bitplane USA; Oxford Instruments). Image analysis by FIJI ImageJ and QuPath was as previously described (28, 29). Following Imaris processing, images were analyzed using FIJI ImageJ version 2.16.0/1.54p (NIH, Bethesda, MD, USA) for the number of immunostained colocalization and cell-cell interactions by using the FIJI Cell Counter plugin. Image analysis was conducted on acquired images as follows: two sections per slide per animal of *n* = 2–3 animals.

## RESULTS

### Kinetics and enrichment dynamics of multiple key immune cell populations in the lung differ drastically during allergic sensitization compared to breakthrough influenza infection

We have established a breakthrough influenza infection model using the 2005–2006 trivalent inactivated influenza vaccine (TIV; Fluzone) as the model vaccine: mice are vaccinated with TIV, then intranasally challenged with a sublethal (0.2 LD_50_) dose of a vaccine-matched influenza virus, H1N1 A/New Caledonia/20/1999 (NC99). Previously, we have shown that the replicating virus is observed early after challenge at 2–3 DPC but is rapidly cleared and undetectable by 7 DPC in vaccinated hosts, in stark contrast to mice without prior vaccination undergoing primary infection, which do not clear the infection by 7 DPC and still have detectable viral titers (23, 24).

We previously observed a twofold to fourfold increase in lung eosinophil numbers in mice experiencing breakthrough infection, with a marked enrichment in the Siglec-F^hi^ subset (24). This lung eosinophilia was not accompanied by an overt Th2 cytokine module, substantial host weight loss, abrogation of alveolar macrophage (AM) numbers, or high concentrations of total serum IgE, unlike what is observed in typical, pathological Type 2 responses such as allergy or VAERD. This non-pathological lung eosinophilia was also present irrespective of adjuvant: inclusion of a Th2-skewing agent, such as alum, or a potent Th1-skewing agent, such as an amphiphilic conjugate of the imidazoquinoline TLR7/8 agonist connected to poly(ethylene glycol) and cholesterol (IMDQ) during vaccination elicited similar numbers of lung eosinophils upon breakthrough infection. This eosinophil recruitment also appeared to be sex-independent, as it was observed in male mice as well (24).

**Fig 1:**
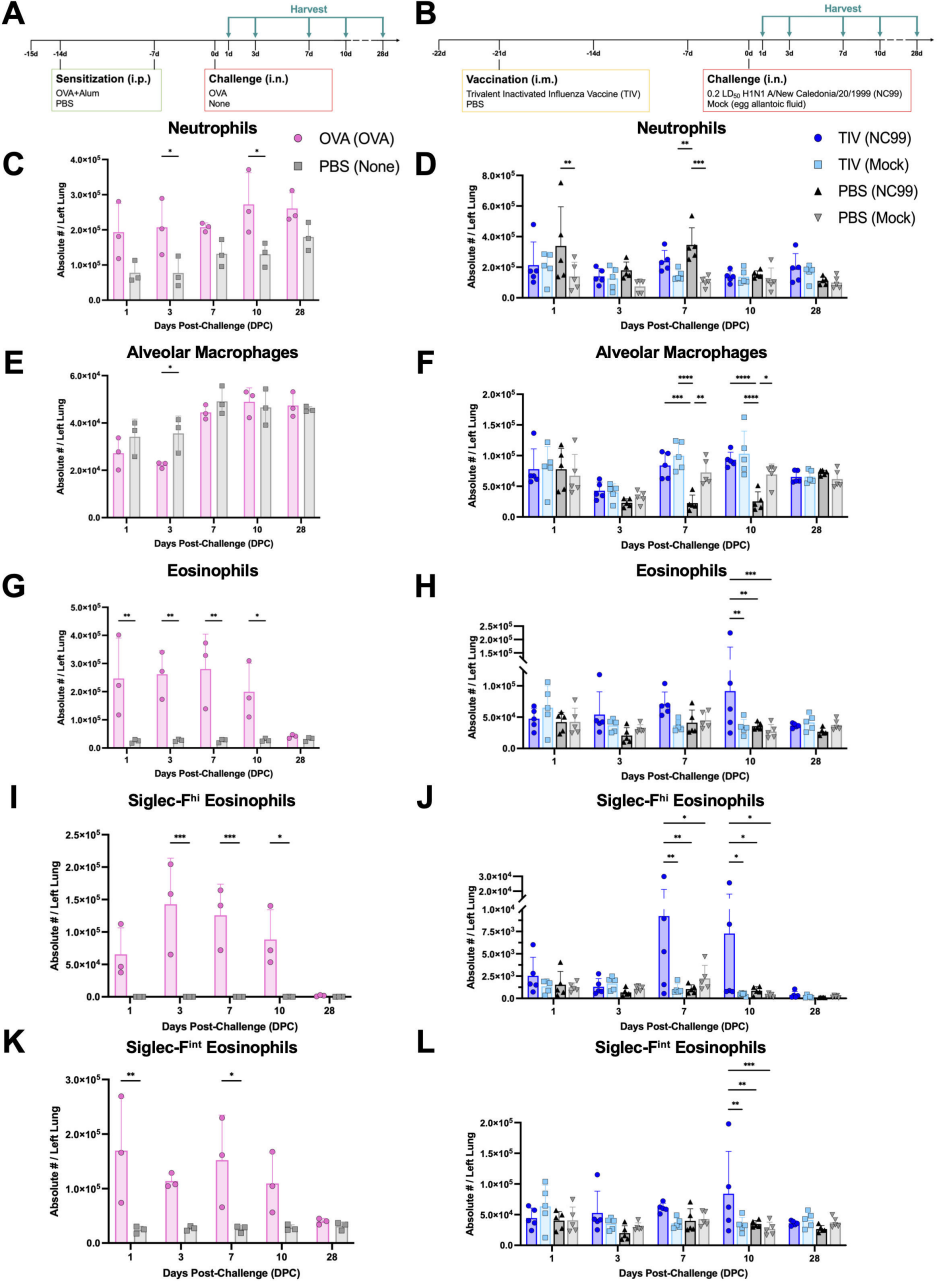
OVA sensitization and breakthrough influenza infection exhibit different immune cell kinetics during acute lung inflammation. Study design for (**A**) OVA sensitization and (**B**) breakthrough infection longitudinal studies. Absolute numbers of (**C, D**) neutrophils (Ly6G^+^), (**E, F**) alveolar macrophages (Ly6G^-^ CD11b^-^ CD11c^+^ Siglec-F^+^ MHC II^hi^), (**G, H**) total eosinophils (Ly6G^-^ CD11b^+^ CD11c^-^ CD125^int^ Siglec-F^+^), (**I, J**) Siglec-F^hi^ eosinophils, and (**K, L**) Siglec-F^int^ eosinophils quantified from the (**C, E, G, I, K**) OVA sensitization or (**D, F, H, J, L**) breakthrough infection longitudinal studies. Bar plots and error bars represent mean ± SD, with each symbol representing an individual mouse. Statistical significance was determined via ordinary two-way ANOVA (**C, E, G, I, K**) with Šídák’s multiple comparisons test with a single pooled variance or (**D, F, H, J, L**) with Tukey’s multiple comparisons test with a single pooled variance. ****P < 0.0001, ***P = 0.0001–0.001, **P = 0.001–0.01, *P = 0.01–0.05.

Thus far, all characterization of immunity has been conducted at 7 days post-challenge (DPC). We expanded our analyses to include additional time points to pinpoint when peak eosinophilia occurs during the acute phase of inflammation and to observe if there are any clear cytokine and chemokine recruitment signals. We also included a later harvest time point at 28 DPC to evaluate whether the inflammatory phenotypic shift of the lung eosinophil population is more long-lived than the reported half-life of 1.5–8 days in the lung, which could point to prolonged recruitment, retention, or local renewal (14, 30, 31). To better understand differences in lung immune cell kinetics, we compared and contrasted a typical, pathological Type 2 response model, ovalbumin (OVA) sensitization, with our experimental breakthrough influenza infection model at days 1, 3, 7, 10, and 28 DPC (Fig. 1A and B). Using flow cytometry, we quantified multiple key immune cell populations in the lung: neutrophils, alveolar macrophages, and eosinophils. The gating strategy is as described in Fig. S1.

Neutrophils are key innate immune cells that can rapidly respond to inflammatory insults. Neutrophil recruitment to the lungs has been implicated in both allergic asthma and respiratory viral infections, with differing effects on host outcome (32, 33). Given their importance in the lung immune response, we quantified total neutrophil counts in both the OVA sensitization model and breakthrough infection model (Fig. 1C and D). In OVA-sensitized mice, a 1.5- to 3.2-fold enrichment in neutrophil count was observed through 10 DPC compared to the PBS control group that received no intranasal challenge, with significant differences at 3 and 10 DPC (Fig. 1C). Lung neutrophil numbers were most elevated at 10 DPC in the OVA sensitized group and sustained, even at 28 DPC, compared to PBS control group which had steadily increasing neutrophil counts through the end of the study. Mice that received PBS vaccination and were challenged with NC99 H1N1 virus, essentially undergoing a primary influenza infection, had significantly higher counts of lung neutrophils at 1 and 7 DPC compared to the PBS-vaccinated mock-challenged controls (Fig. 1D). The TIV-vaccinated NC99-challenged group, which modeled a breakthrough infection, had relatively stable numbers of lung neutrophils throughout the course of the study, similar to the TIV-vaccinated mock-challenged group and PBS-vaccinated mock-challenged group. Within the two TIV-vaccinated groups, neutrophil counts were not significantly different, irrespective of whether the mice were challenged or not.

Next, we quantified the number of AMs in the lungs of mice undergoing OVA sensitization or breakthrough infection (Fig. 1E and F). AMs are tissue-resident macrophages in the alveolar space and have a multitude of homeostatic and inflammatory functions. AMs serve as the first line of defense against perturbations in the respiratory tract and display functional plasticity, capable of promoting both pro- and anti-inflammatory states in allergic asthma, respiratory viral infections, and more (34, 35). In OVA-sensitized mice, the number of lung AMs was significantly reduced at 3 DPC compared to PBS control mice, although counts rapidly recovered to the level of control mice by 10 DPC (Fig. 1E). This is in line with findings demonstrating that AMs can die during airway allergic responses (36). In the breakthrough infection model, both the NC99- and mock-challenged groups, irrespective of vaccine regimen, had lower numbers of AMs in the lung at 3 DPC relative to 1 DPC (Fig. 1F). However, for all groups except for PBS-vaccinated NC99-challenged primary influenza infection mice, AM numbers were similar to that of the unchallenged PBS-negative control in the OVA sensitization model, suggesting that the counts at 3 DPC, while lower than 1 DPC, are still within the homeostatic range or were transiently reduced as a result of the intranasal instillation of challenge material (Fig. 1E). AM count rapidly increased by 7 DPC for all groups in the breakthrough infection model, except for the PBS-vaccinated NC99-challenged primary infection mice, which had a highly significant reduction in AM numbers through 10 DPC. In contrast, TIV-vaccinated NC99-challenged breakthrough infection mice had no such reduction in AMs, with numbers resembling those of the TIV-vaccinated mock-challenged healthy controls at all time points in the study. This corroborates previous studies, where we and others have demonstrated that vaccine-mediated immunity prevents AM death upon subsequent influenza infection (23, 24, 37–40). By 28 DPC, all groups had equivalent numbers of AMs.

Eosinophils are recruited during Type 2 responses, such as allergy and asthma, where eosinophil degranulation or release of cytokines can facilitate immunopathologic disease (30, 41, 42). Lung eosinophil influx has also been associated with respiratory viral breakthrough infection, particularly in the case of RSV VAERD, although they are not directly responsible for the enhanced disease phenotype; Th2 CD4 T cells are the main drivers of RSV VAERD (17–19). Eosinophils during influenza breakthrough infection have been investigated by our group, and in contrast with observations in the RSV model and allergy models, lung eosinophils do not correlate with enhanced disease, but rather with protection (23, 24). As such, we were interested in quantifying the number of lung eosinophils following OVA allergic sensitization and comparing it with the breakthrough infection model to understand the differences in kinetics, magnitude of recruitment, and phenotype (Fig. 1G and H).

After the OVA challenge, all OVA-sensitized mice had a substantial enrichment in lung eosinophil count compared to the PBS control group during the acute phase of inflammation (Fig. 1G). OVA-sensitized mice had 9.6- to 10.7-fold more eosinophils than the PBS control at 1-7 DPC. By 10 DPC, the number of eosinophils was lower than in earlier time points, although there were still 7.1-fold more eosinophils in the OVA-sensitized group than the PBS controls. By 28 DPC, the eosinophil numbers were not significantly different between the two groups. For the breakthrough infection model, the TIV-vaccinated NC99-challenged breakthrough infection group generally had the highest numbers of lung eosinophils compared to other groups during the acute stage of infection (Fig. 1H). Eosinophil counts were 2.6-fold higher in the breakthrough infection group compared to the primary infection group at 3 DPC. There was a 1.8-fold enrichment in eosinophils in the breakthrough infection group that received a sublethal NC99 challenge, compared to the mock-challenged controls at 7 DPC. This is in line with previous experiments we conducted at 7 DPC (24). The breakthrough infection group also had a statistically significant, 1.6- to 2.2-fold greater enrichment in eosinophil numbers at 10 DPC relative to other groups. By 28 DPC, all four treatment groups had similar numbers of eosinophils. In terms of kinetics, only the breakthrough infection group had a distinct rise-fall in eosinophil numbers, peaking at 10 DPC, while all other groups were relatively stable in eosinophil counts throughout the entire study.

Within the lung eosinophil population, we quantified two subpopulations: Siglec-F^hi^ eosinophils and Siglec-F^int^ eosinophils. Siglec-F^hi^ eosinophils are thought to be the more activated, inflammatory subset, while Siglec-F^int^ eosinophils have been described to be the more resting, homeostatic phenotype in the lungs (25). CD101 expression has also been used to subset eosinophils, with the CD101^+^ eosinophils corresponding to more pro-inflammatory functions and prevalent in pathological Type 2 responses, such as allergy and helminth infections (25). CD101^+^ eosinophils have also been observed during breakthrough infection by our group, although they did not appear to correlate with host pathology (24). We observed that 75-90% of the Siglec-F^hi^ eosinophils were CD101^+^ at any given time point, in alignment with the literature (Fig. S2A and B) (25, 43–45). In the OVA-sensitized mice, Siglec-F^hi^ eosinophils were robustly recruited to the lungs 1-10 DPC, whereas this subset of eosinophils did not have a substantial presence in PBS control mice that received no intranasal challenge throughout the entire study (Fig. 1I). Numbers of this subset peaked in the OVA-sensitized mice at 3 DPC and gradually began to fall through 10 DPC. Although Siglec-F^hi^ eosinophil numbers were largely reduced by 28 DPC, OVA-sensitized mice still had 9.8-fold higher Siglec-F^hi^ eosinophils in the lung compared to the PBS control mice. Siglec-F^hi^ eosinophils were also the majority of the total eosinophil population 3–10 DPC in the OVA-sensitized mice, indicative of a phenotypic shift toward or preference for recruitment of this subset during acute allergic inflammation (Fig. S3A). For the breakthrough infection model, we observed that all groups had some degree of recruitment of Siglec-F^hi^ eosinophils to the lung after any intranasal challenge (Fig. 1J). This subset was most prevalent in the breakthrough infection group alone, with significantly higher numbers than all other treatment groups at 7 and 10 DPC. Similar to kinetics seen in the OVA-sensitization study, the number of Siglec-F^hi^ eosinophils was much lower in all groups by 28 DPC compared to earlier time points in the study. Of note, the number of Siglec-F^hi^ eosinophils was >2-fold higher in the TIV-vaccinated NC99-challenged breakthrough infection mice compared to all other groups at 28 DPC, even at this later time point. Although Siglec-F^hi^ eosinophils were never the majority of the total eosinophil population for any of the treatment groups in the breakthrough infection model, this subset was significantly enriched at 7 DPC in the breakthrough infection group only: Siglec-F^hi^ eosinophils were 10.8% of eosinophils for TIV-vaccinated NC99-challenged mice compared to the TIV-vaccinated mock-challenged (2.9%), PBS-vaccinated NC99-challenged (4.7%), and PBS-vaccinated mock-challenged (2.7%) groups (Fig. S3B). Mirroring observations in Siglec-F^hi^ eosinophil counts, the frequency of this subset remained elevated in only the breakthrough infection group (1.1%) at 28 DPC, while <1% of total eosinophils were Siglec-F^hi^ eosinophils in all other groups by this time point.

Next, we quantified the number of Siglec-F^int^ eosinophils in the lungs for both models (Fig. 1K and L). In the OVA-sensitization model, numbers of Siglec-F^int^ eosinophils were elevated from 1 to 10 DPC and were the highest on 1 and 7 DPC (Fig. 1K). In contrast, PBS control mice had relatively stable numbers of Siglec-F^int^ eosinophils throughout the entire study. Unlike what was seen for the Siglec-F^hi^ eosinophils, where numbers were still elevated above the control group at 28 DPC, the numbers of Siglec-F^int^ eosinophils were similar to those of the controls by this later time point. Although there was also a dramatic increase in the numbers of this subset, Siglec-F^int^ eosinophils represented <50% of total eosinophils at 3–10 DPC due to the increase in Siglec-F^hi^ eosinophils in the OVA-sensitized mice. Otherwise, in PBS control mice that received no intranasal challenge, >99% of eosinophils have the Siglec-F^int^ phenotype (Fig. S3C). In the breakthrough infection model, Siglec-F^int^ kinetics for each treatment group largely resembled that of the total eosinophil population (Fig. 1H and L). The enrichment in Siglec-F^int^ eosinophils was most apparent at 7 and 10 DPC: the TIV-vaccinated NC99-challenged breakthrough infection group had, respectively, 1.7- or 2.5-fold higher counts of Siglec-F^int^ eosinophils than the TIV-vaccinated mock-challenged control group. Breakthrough infection had significantly greater numbers of this subset than all other groups at 10 DPC. By 28 DPC, all groups had similar numbers of Siglec-F^int^ eosinophils, unlike what was observed for the Siglec-F^hi^ subset. We saw that Siglec-F^int^ eosinophils represented less of the total eosinophils at 7 DPC, corresponding to the significantly increased frequency of Siglec-F^hi^ eosinophils (Fig. S3D).

In summary, drastically different immune cell population dynamics were observed for both models used in this study. OVA sensitization presented with the hallmarks of pathogenic Type 2 immune responses: exuberant lung eosinophilia for both subsets, transient loss of AMs, and enrichment in neutrophilic inflammation as well. In contrast, primary influenza infection in hosts without pre-existing vaccine immunity exhibited neutrophilia, marked and sustained loss of AMs, but no eosinophilia. Breakthrough infection of vaccinated hosts following sublethal viral challenge presented with a lung cellular composition that had elements of both the OVA-sensitized, Type 2 response and the primary influenza infection Type 1 response: mild neutrophil enrichment was observed, relative to vaccinated and mock-challenged controls, but not to the same extent as primary influenza infection mice; no dramatic loss of AMs was observed; and total eosinophil counts were significantly higher in this group at 7–10 DPC, with a substantial enrichment for the Siglec-F^hi^ subset, although not to the same extent as the OVA-sensitized mice.

### OVA-sensitized allergic mice and unvaccinated influenza-infected mice have exuberant pro-inflammatory cytokine and chemokine expression in the lung during the acute phase of inflammation

**Fig 2:**
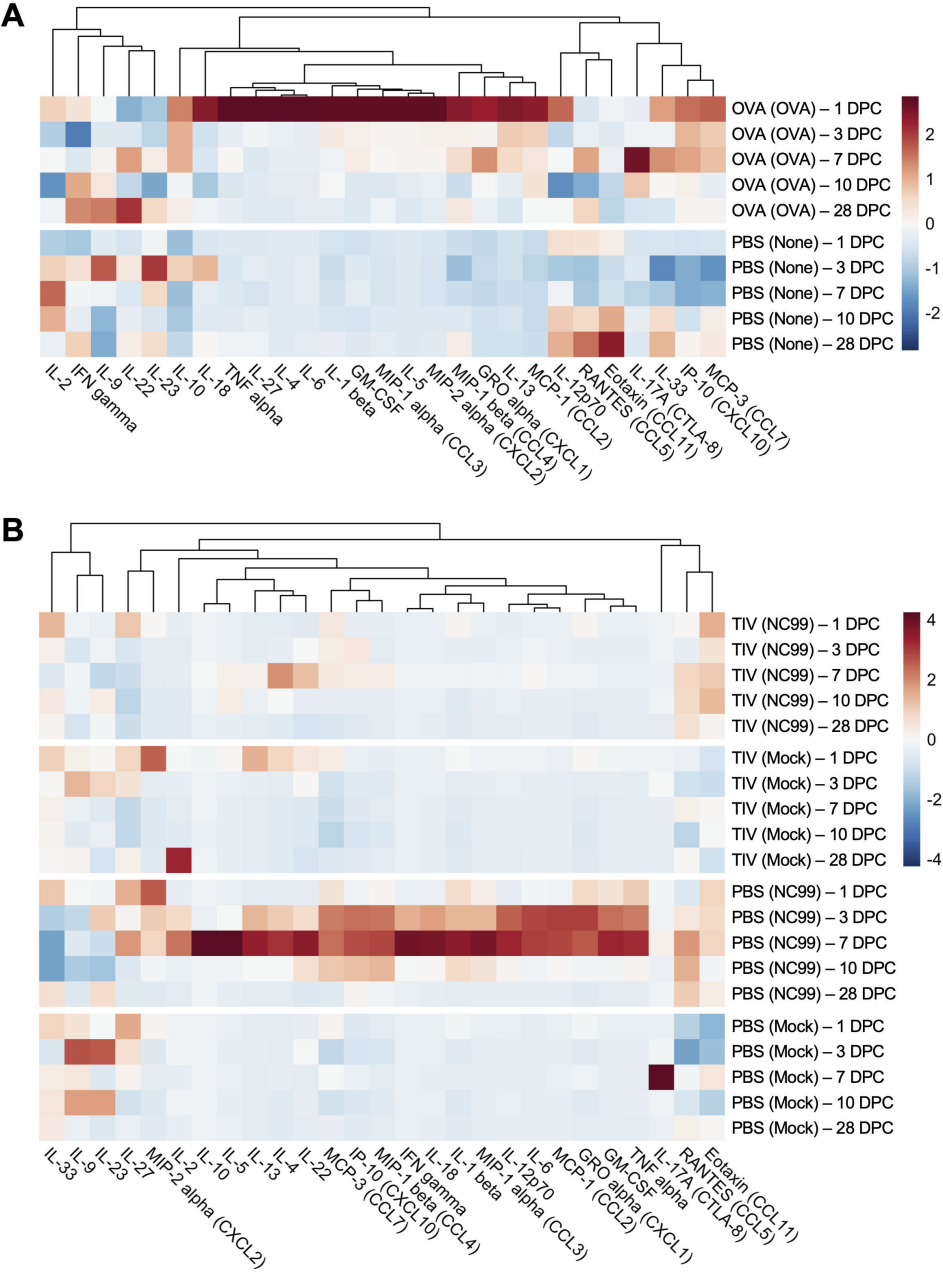
A breakthrough infection does not exhibit a distinctly Th1 or Th2 lung cytokine and chemokine module during acute and late infection, unlike OVA sensitization and primary influenza infection. Heat map of net mean fluorescence intensity (MFI) of each analyte, *z*-scored by column. (**A**) OVA-sensitized mice and controls and (**B**) breakthrough infection mice and controls. Each square represents the average net MFI for the treatment group at that time point (*n* = 3–5).

As a snapshot of the inflammatory profile of the lungs, we next measured the concentrations of 27 different cytokines and chemokines in lung homogenate supernatants for both models. In the OVA-sensitization study mice, we saw a very distinct pro-inflammatory cytokine and chemokine profile most prevalent at 1 DPC in the OVA-sensitized mice, which was not observed in the PBS control mice that did not receive an intranasal challenge (Fig. 2A; Fig. S4). OVA-sensitized mice had high concentrations of IL-1β, IL-6, IL-18, IL-27, GM-CSF, CCL2, CCL3, CCL4, CXCL1, and CXCL2. Type 1 cytokines such as TNF-α and IL-12, but not IFN-γ, were also substantially elevated in OVA-sensitized mice at 1 DPC but not in PBS control mice. Canonical Type 2 cytokines such as IL-4, IL-5, and IL-13 were significantly higher at 1 DPC in the OVA-sensitized mice and remained higher than concentrations observed in the PBS control mice through 7 DPC. Interestingly, CCL11 (Eotaxin-1) did not appear to have a strong signal at the acute time points when peak lung eosinophilia was observed in OVA-sensitized mice.

Distinct cytokine and chemokine profiles were observed in the breakthrough infection model, contrasting with observations in the OVA-sensitized mice (Fig. 2B; Fig. S5). Many pro-inflammatory cytokines and chemokines were highly expressed in the PBS-vaccinated NC99-challenged primary infection mice at 3 and 7 DPC at significantly higher concentrations than all other groups: IL-1β, IL-6, IL-18, IL-22, IL-27, CCL2, CCL3, CXCL2, CCL4, CCL5, CCL7, CXCL1, and CXCL10. Canonical Type 1-associated cytokines, such as IFN-γ, TNF-α, IL-2, and IL-12, were most prominent in this group. This is in line with expectations since this group was undergoing a primary viral infection. Additionally, Type 2 cytokines such as IL-4 and IL-13 were also present in the primary infection mice at these two time points, and with significantly higher IL-5 expression at 7 DPC than all other groups. Anti-inflammatory cytokine IL-10 was most highly expressed at 7 DPC in primary infection mice. Concentrations of the alarmin IL-33 were depressed in the primary infection mice most prevalently at days 3–10 post-challenge, ultimately recovering to the levels of all other treatment groups by 28 DPC. In stark contrast, the TIV-vaccinated NC99-challenged breakthrough infection mice had a substantially less inflammatory cytokine and chemokine profile in the lungs compared to primary infection mice. Type 1 cytokines were slightly elevated in TIV-vaccinated NC99-challenged breakthrough infection mice compared to TIV-vaccinated mock-challenged controls, but still significantly lower when compared to the PBS-vaccinated NC99-challenged primary infection mice. Similarly, low levels of Type 2 cytokines were observed in the breakthrough infection mice, but concentrations were still significantly higher in the primary infection mice. Concentrations of IL-4 were higher in the breakthrough infection mice compared to the mock-challenged controls at 7 DPC, but still significantly lower than the primary infection mice. CCL11 concentrations were significantly elevated in the breakthrough infection mice at 1 DPC compared to the primary infection mice, but otherwise were at similar concentrations as all other treatment groups at the remainder of the time points. Of note, peak CCL11 concentrations preceded the peak lung eosinophilia, observed at 7–10 DPC, by several days in the breakthrough infection mice. IL-27 concentrations in the breakthrough infection mice were significantly lower than those of PBS-vaccinated mock-challenged controls at 3 DPC, and significantly lower than those of TIV-vaccinated mock-challenged controls at 28 DPC.

Complementing our cellular quantification, we observed a distinct Type 2 cytokine and chemokine module for OVA-sensitized mice, a clear Type 1 profile for primary infection mice, and a more balanced, muted inflammatory profile for the breakthrough infection mice. Peak cytokine and chemokine signatures were observed at more acute time points following intranasal challenge, such as 1 DPC for the OVA-sensitized mice and 3–7 DPC for the primary infection mice.

### Host morbidity corresponds with viral titers for breakthrough and primary influenza infection in mice

**Fig 3:**
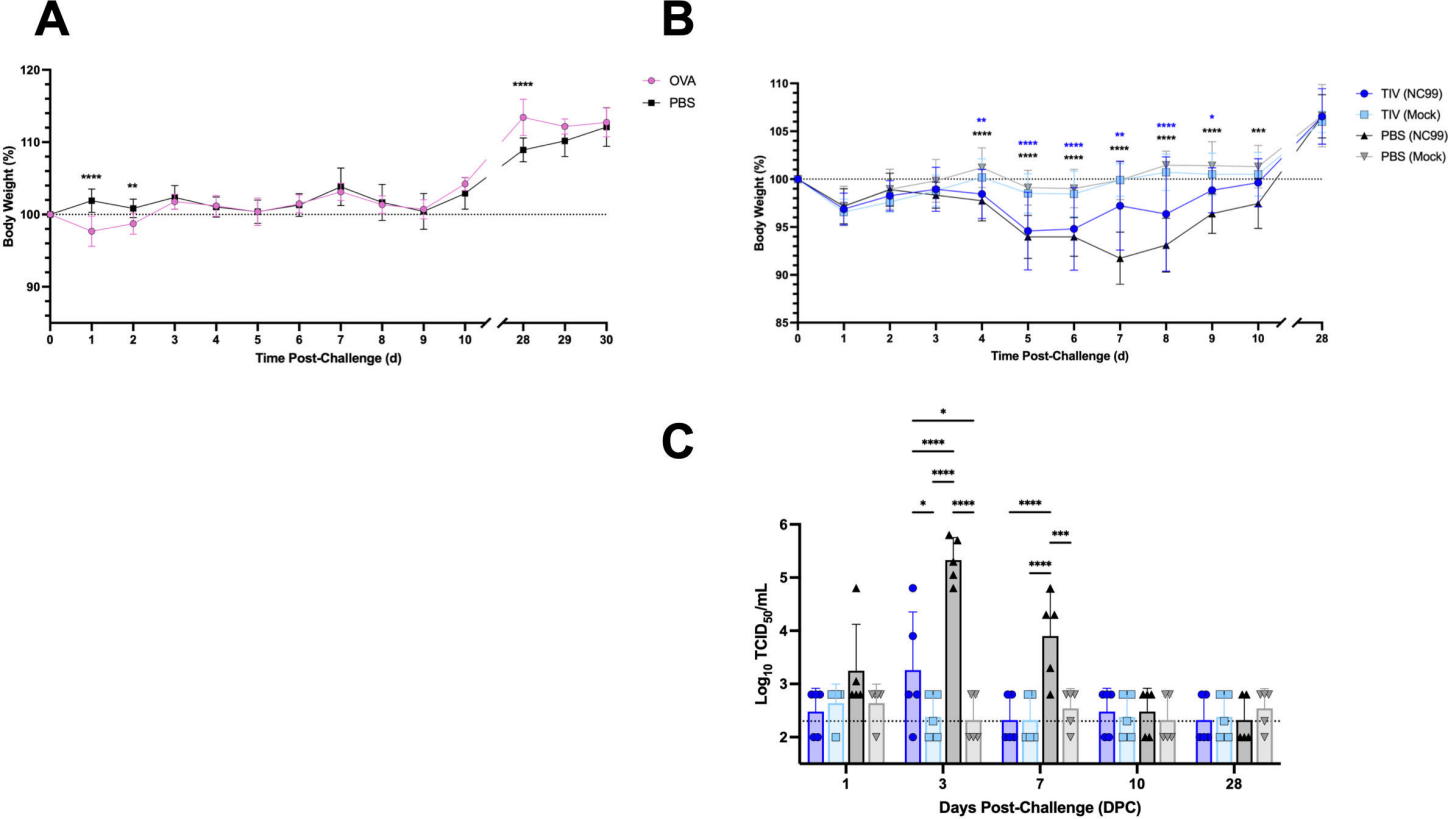
Host body weight does not correlate to lung immune inflammation in OVA-sensitized mice, but does reflect the severity of viral load in mice experiencing breakthrough or primary influenza infection. Morbidity for (**A**) OVA-sensitized mice and controls or (**B**) breakthrough infection mice and controls. (**C**) Replicating viral load in the lungs was determined via TCID_50_ for breakthrough infection mice and controls. Dotted lines denote (**A, B**) 100% body weight or (**C**) LOD of 200 TCID_50_/mL. Bar plots and error bars represent mean ± SD, with each symbol representing an individual mouse. Statistical significance was determined via ordinary two-way ANOVA with (**A**) Šídák’s multiple comparisons test with a single pooled variance, (**B**) Dunnett’s multiple comparisons test with a single pooled variance using the PBS (Mock) group as the comparator for all other groups, or (**C**) Tukey’s multiple comparisons test, with a single pooled variance. Asterisks are color-coded by group in (**B**). ****P < 0.0001, ***P = 0.0001–0.001, **P = 0.001–0.01, *P = 0.01–0.05.

To bolster our understanding of general host health status beyond lung immunity, we used body weight as a metric of morbidity for both models. Despite exuberant recruitment of eosinophils to the lung, compounded by neutrophilia and transient loss of alveolar macrophages, OVA-sensitized mice did not have drastic reductions in body weight, unlike what is observed during severe respiratory viral infection (Fig. 3A). There was statistically significant weight loss on 1–2 DPC, with OVA-sensitized mice losing < 3% body weight while PBS control mice did not. However, this difference may be due to sedation since PBS control mice received no intranasal challenge and therefore were not sedated. At 28 DPC, OVA-challenged mice had significantly higher body weights than the PBS control mice.

For the breakthrough infection study, both the TIV- and PBS-vaccinated groups that received the sublethal NC99 challenge lost weight (Fig. 3B). PBS-vaccinated NC99-challenged primary infection mice continually lost weight through 7 DPC before recovering, with a maximum loss of 8.3% body weight at 7 DPC. In contrast, TIV-vaccinated NC99-challenged breakthrough infection mice began to recover at 5 DPC and had a maximum weight loss of 5.4% at this time point. By 10 DPC, breakthrough infection mice had similar weights as the PBS-vaccinated mock-challenged control group, whereas primary infection mice still had significantly lower body weights. By 28 DPC, both virus-challenged groups had equivalent body weights as the mock-challenged control groups. Our morbidity data were corroborated by our viral load measurements. We found that primary infection mice had the highest viral burden at 3 DPC, significantly higher than all other groups, including mice with vaccine protection (Fig. 3C). There was a low level of replicating virus measured in the lungs of TIV-vaccinated NC99-challenged mice at 3 DPC, confirming breakthrough infection. By 7 DPC, breakthrough infection mice had TCID_50_ measurements equivalent to the mock-challenged groups, indicating rapid control of the virus. In contrast, primary infection mice that received PBS vaccination and had no prior immunity against influenza still had significantly higher viral load at 7 DPC when compared to all other groups. By 10 DPC, primary infection mice had TCID_50_ measurements similar to the TIV-vaccinated NC99-challenged group as well as the mock-challenged groups, indicating control of viral replication by this time point.

### Lung disease is most severe for OVA-sensitized mice and primary influenza infection mice at acute time points, while breakthrough infection mice are protected from severe disease and do not exhibit extensive mucin staining

**Fig 4:**
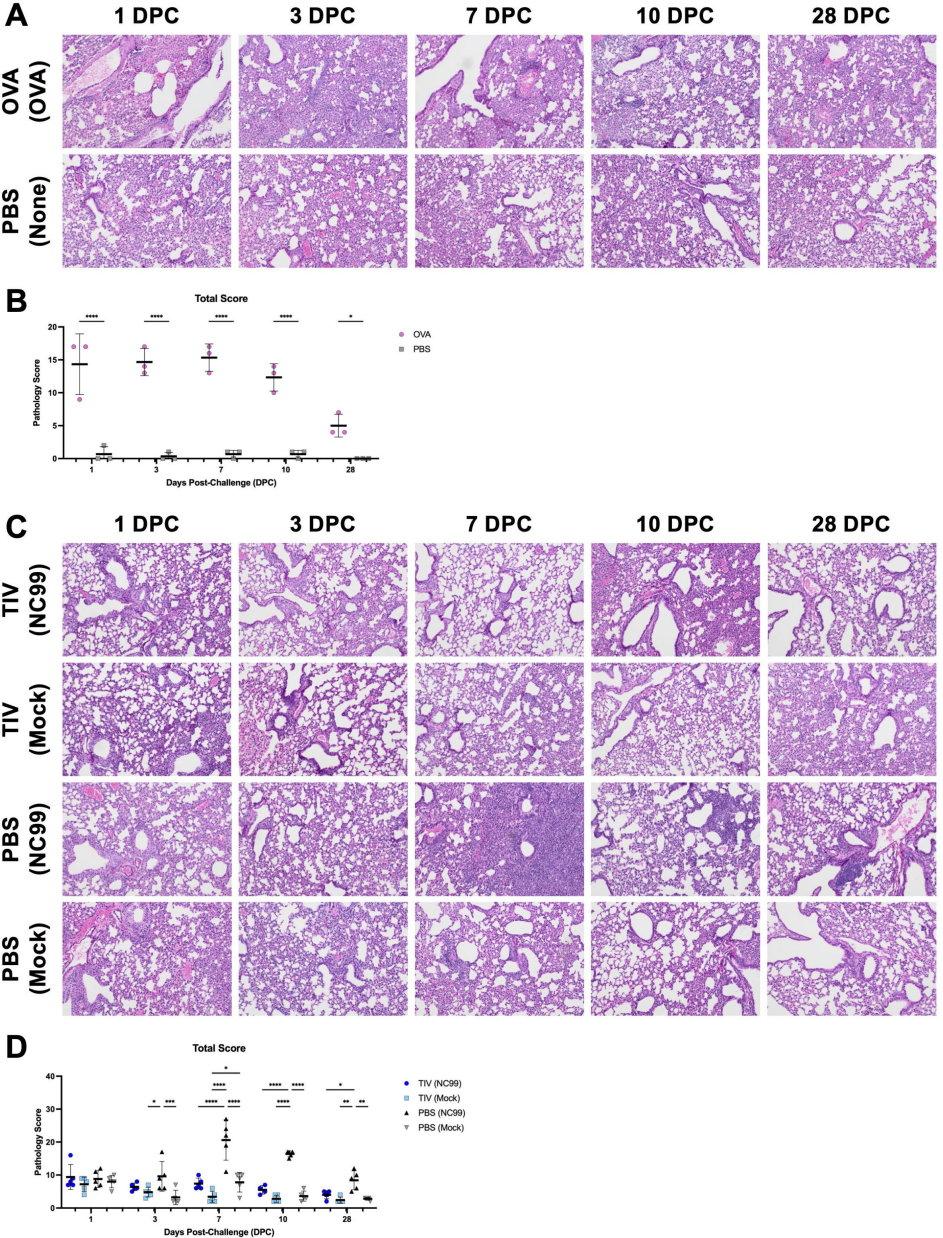
A breakthrough infection exhibits less disease compared to OVA allergic sensitization or primary influenza infection. Lung sections were stained with H&E and then subjected to blinded pathology scoring. (**A**) Representative images and (**B**) total pathology score for the OVA sensitization model. (**C**) Representative images and (**D**) total pathology score for the breakthrough infection model. The total pathology score was comprised of the sum of the following metrics, individually scored from a scale of 1 to 5+: amount of lung affected and perivascular inflammation within the total lung; neutrophil inflammation, eosinophil inflammation, basophil inflammation, mast cell inflammation, lymphocyte/plasma cell inflammation, epithelial necrosis, intraluminal cells or debris, bronchus-associated lymphoid tissue (BALT) hyperplasia in the bronchi and bronchioles or peribronchial and peribronchiolar areas; neutrophil inflammation, eosinophil inflammation, basophil inflammation, mast cell inflammation, lymphocyte/plasma cell/macrophage inflammation, necrosis/fibrin, consolidation, and edema in the alveoli or alveolar septa. Lines and error bars represent mean ± SD, with each symbol representing an individual mouse. Statistical significance was determined via ordinary two-way ANOVA with (**B**) Šídák’s multiple comparisons test with a single pooled variance, or (**D**) Tukey’s multiple comparisons test with a single pooled variance. ****P < 0.0001, ***P = 0.0001–0.001, **P = 0.001–0.01, *P = 0.01–0.05.

After quantifying lung immune cell populations, inflammatory cytokine and chemokine concentrations, viral load, and overall host morbidity, we sought to assess the severity of lung lesions as well (Fig. 4A through D). Histopathological analyses revealed extensive histopathologic changes in the lungs of OVA-sensitized mice compared to PBS controls, most prominent during the acute phase of inflammation (Fig. 4A and B; Fig. S6). Significant lesions were observed from 1 DPC through 7 DPC, before decreasing at 10 DPC. OVA-sensitized mice still had significantly higher total pathology scores compared to control mice at 28 DPC. Extensive neutrophil, eosinophil, lymphocyte, plasma cell, and macrophage inflammation were observed in OVA-sensitized mice, corroborating flow cytometry results (Fig. S6B and C). Mast cells and basophils were not substantially observed, likely due to cell rarity and the specific portion of the lung evaluated; mast cells are more prevalent in the upper respiratory tract rather than the lower respiratory tract, such as the lung lobes.

Severe lung lesions were observed in the PBS-vaccinated NC99-challenged primary infection mice when compared to other groups, such as the breakthrough infection mice and mock-challenged controls (Fig. 4C and D; Fig. S7). General patterns for neutrophil, eosinophil, and macrophage inflammation observed in histopathological analyses were in line with findings from flow cytometry (Fig. S7B and C). Scores for eosinophilic inflammation were lower for mice in the breakthrough infection model compared to the OVA-sensitization model. Primary infection mice had severe disease, unlike the breakthrough infection mice, with significantly higher total scores than all other groups at 7–28 DPC. Epithelial necrosis, as well as intraluminal cells and debris, was most prevalent in primary infection mice compared to breakthrough infection mice and mock-challenged controls (Fig. S7B). Bronchus-associated lymphoid tissue (BALT) hyperplasia was only observed in primary infection mice, beginning from 7 DPC and scores steadily increased through 28 DPC (Fig. S7B). Primary infection mice also had substantial consolidation, necrosis, and fibrin in the alveoli and alveolar septa at 7–10 DPC, while other groups did not score highly for these metrics (Fig. S7C). Breakthrough infection mice that received vaccination and had a lower viral load did have some histopathologic changes, but not to the same extent as primary infection mice: higher perivascular inflammation and epithelial necrosis were observed in TIV-vaccinated NC99-challenged breakthrough infection mice relative to TIV-vaccinated mock-challenged controls, particularly at 1–10 DPC (Fig. S7A and B). However, pathology scores were significantly higher in the PBS-vaccinated NC99-challenged group compared to the TIV-vaccinated NC99-challenged group for the same metrics.

**Fig 5:**
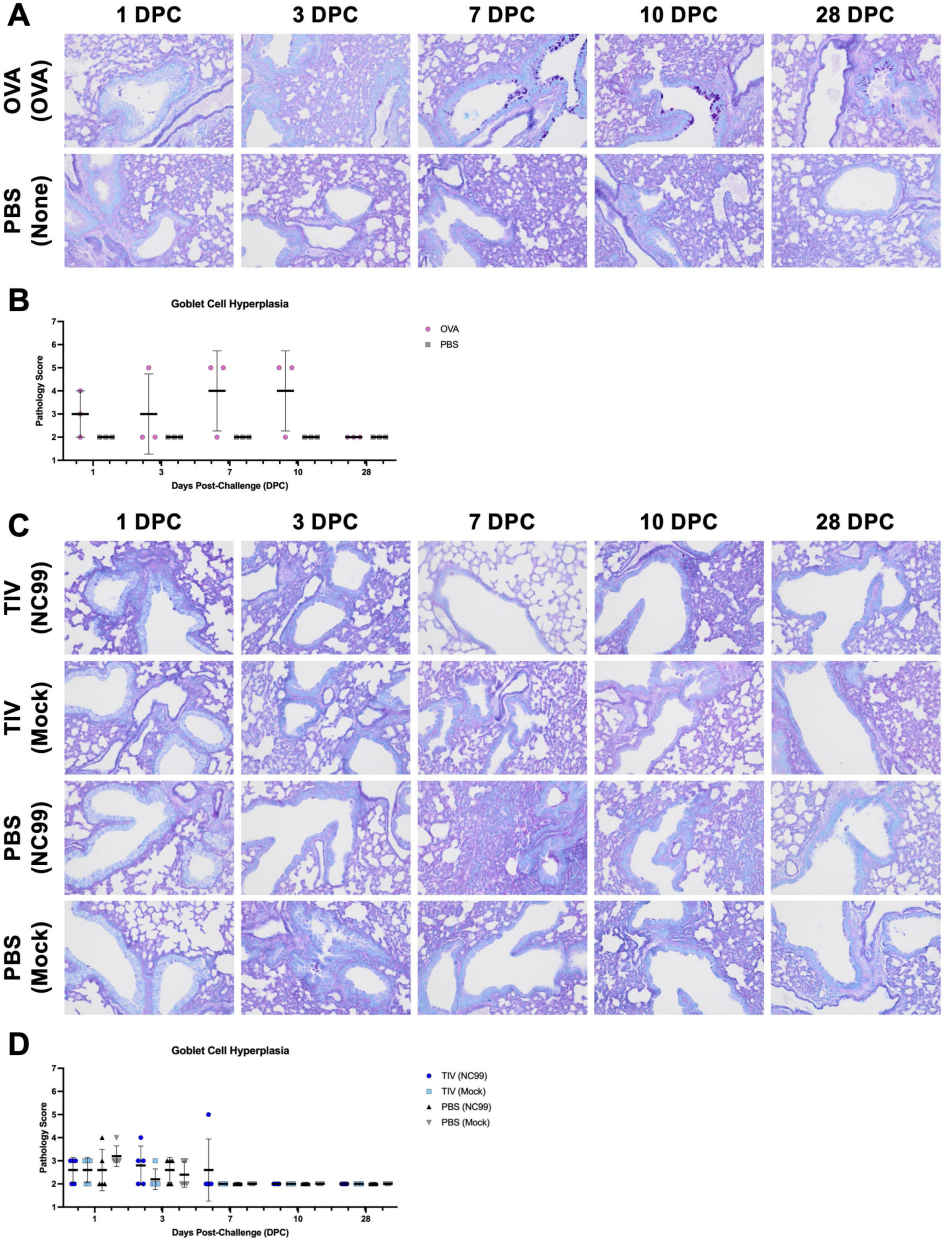
Goblet cell hyperplasia and positive mucin staining are observed during Type 2 allergic responses but not during breakthrough or primary influenza infection. Lung sections were stained with AB-PAS to assess mucin staining and goblet cell hyperplasia, then subjected to blinded pathology scoring. (**A**) Representative images and (**B**) goblet cell score for the OVA sensitization model. (**C**) Representative images and (**D**) goblet cell score for the breakthrough infection model. Lines and error bars represent mean ± SD, with each symbol representing an individual mouse. Statistical significance was determined via ordinary two-way ANOVA with (**B**) Šídák’s multiple comparisons test with a single pooled variance, or (**D**) Tukey’s multiple comparisons test with a single pooled variance.

To better understand host health, we then employed another metric of lung pathology. Excess mucin has been associated with Type 2-skewed disease states, such as allergy, asthma, and RSV VAERD (18). As such, we scored Alcian Blue/Periodic acid–Schiff stained sections to determine the degree of mucin and goblet cell hyperplasia. We observed extensive mucin staining and goblet cell hyperplasia in OVA-sensitized, allergic mice, most prevalently at 7 and 10 DPC (Fig. 5A and B). No mucin staining or goblet cell hyperplasia was observed in PBS controls. No substantial mucin staining or goblet cell hyperplasia was observed for any of the treatment groups in the breakthrough influenza infection model as well (Fig. 5C and D).

### OVA-sensitization and primary influenza infection result in isotype-skewed serum antibody responses, while TIV-vaccination confers a balanced IgG2a/IgG1 profile

**Fig 6:**
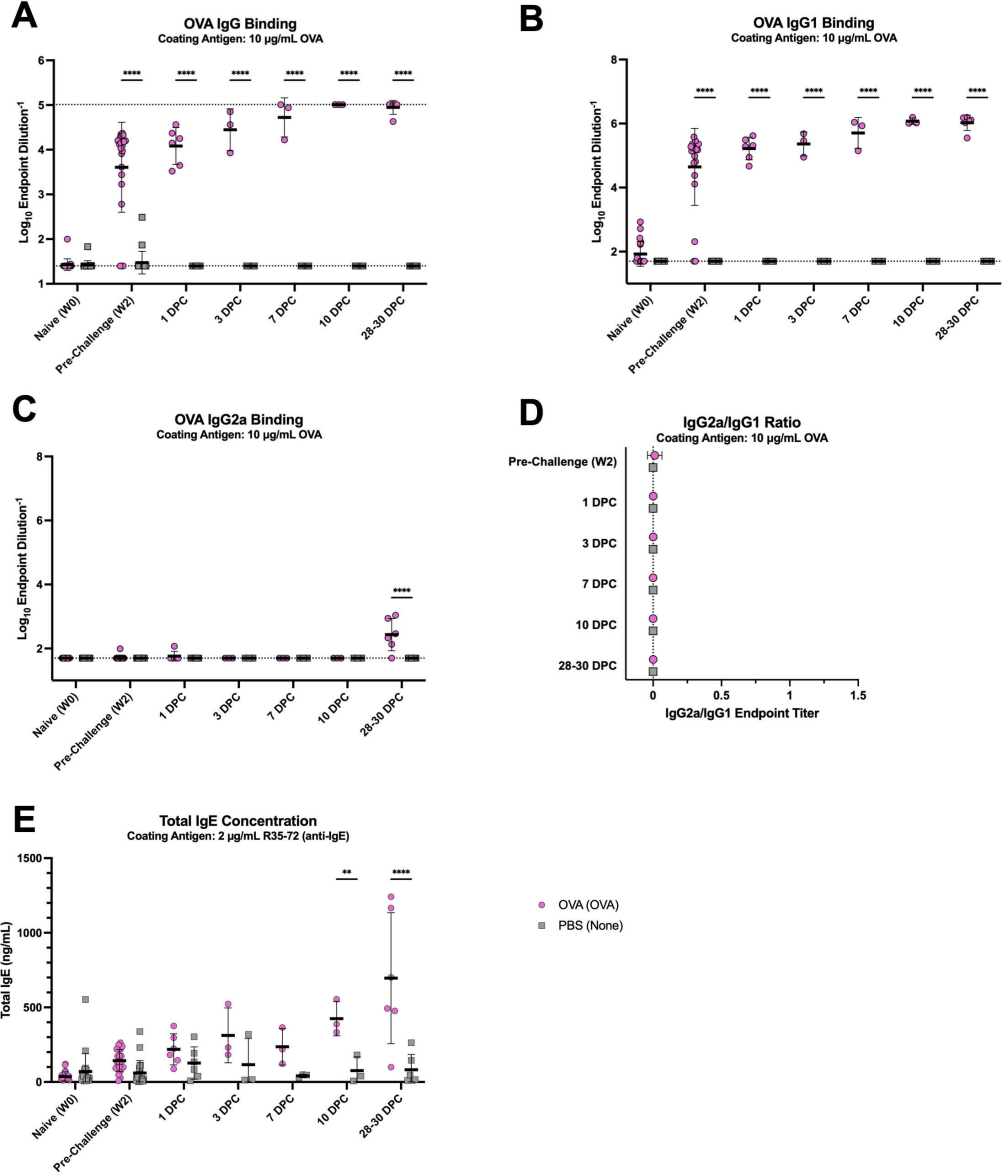
OVA-sensitized mice mount IgG1-biased serum antibody responses with increasing total IgE concentrations after intranasal challenge with antigen. OVA-binding (**A**) total IgG, (**B**) IgG1, and (**C**) IgG2a endpoint titers were measured. The dotted line represents an endpoint titer LOD of (**A**) 25 or (**B–C**) 50. (**A**) Total OVA IgG binding had an upper LOD of 102,400. Undetectable values were arbitrarily set to the LOD. (**D**) The ratio of IgG2a/IgG1 endpoint titers was used to evaluate immune skewing. The dotted line represents a ratio of 0. Mice that had undetectable IgG1 and IgG2a endpoint titers for a given time point were arbitrarily set to a ratio of 0. (**E**) Total serum IgE concentrations were measured. Lines and error bars represent mean ± SD, with each symbol representing an individual mouse. Statistical significance was determined via ordinary two-way ANOVA with Šídák’s multiple comparisons test with a single pooled variance. ****P < 0.0001, **P = 0.001–0.01.

We quantified total antigen-specific IgG, IgG1, and IgG2a titers to evaluate host humoral immunity after vaccination and challenge. In OVA-sensitized mice, we had detectable titers of OVA-specific total IgG by 2 weeks post-vaccination, which continued to steadily rise post-challenge (Fig. 6A). OVA-specific IgG titers were negligible for PBS control mice throughout the entire study. Similar patterns were observed for both treatment groups regarding OVA-specific IgG1 (Fig. 6B). Given that the mice were primed with two intraperitoneal doses of OVA with alum adjuvant, a Type 2-skewing adjuvant (46), we did not expect to see substantial titers of IgG2a in OVA-sensitized mice. Indeed, OVA-specific IgG2a titers were only detectable at 28–30 DPC with intranasal OVA (Fig. 6C). We used the ratio of antigen-specific IgG2a to IgG1 titers as a surrogate for evaluating Th1/Th2-skewing, with higher values indicating more IgG2a- and Th1-biased responses and lower values reflecting IgG1- and Th2-biased responses (47–49). OVA-sensitized mice exhibited very low IgG2a/IgG1 ratios, due to the heavily skewed IgG1 response and in line with the dogmatic Type 2 immune profile in the lung (Fig. 6D). Furthermore, OVA-sensitized mice also generated increasingly higher concentrations of total IgE following intranasal OVA challenge, with peak concentrations observed at 28–30 DPC, significantly greater than concentrations in PBS control mice (Fig. 6E).

**Fig 7:**
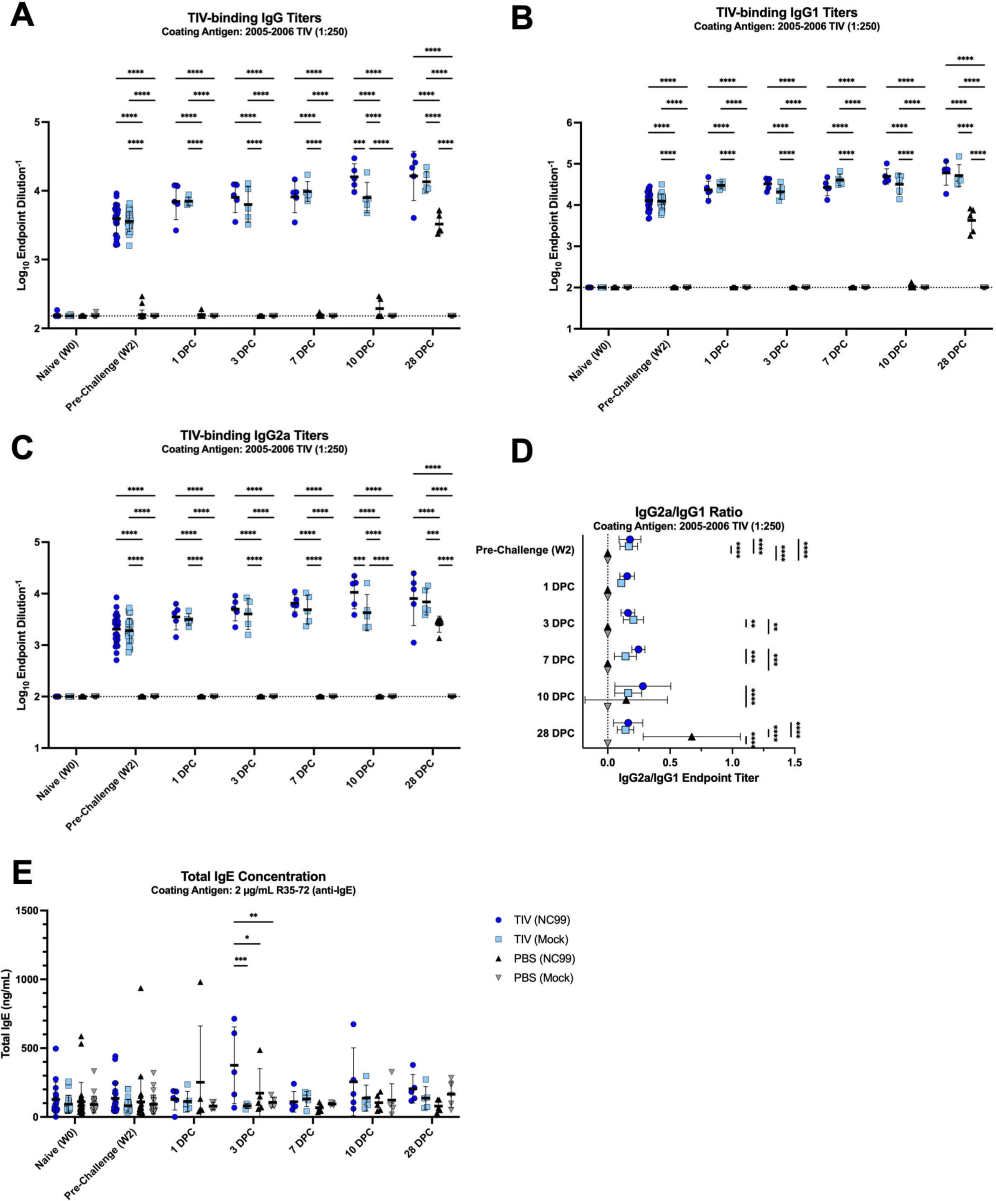
TIV-vaccinated mice seroconvert by 2 weeks post-vaccination and have balanced IgG1 and IgG2a titers with some increase in total IgE concentration shortly after sublethal viral challenge, while primary infection mice have IgG2a-biased responses following challenge. TIV-binding (**A**) total IgG, (**B**) IgG1, and (**C**) IgG2a endpoint titers were measured. The dotted line represents an endpoint titer LOD of (**A–C**) 100. Undetectable values were arbitrarily set to the LOD. (**D**) The ratio of IgG2a/IgG1 endpoint titers was used to evaluate immune skewing. The dotted line represents a ratio of 0. Mice that had undetectable IgG1 and IgG2a endpoint titers for a given time point were arbitrarily set to a ratio of 0. (**E**) Total serum IgE concentrations were measured. Lines and error bars represent mean ± SD, with each symbol representing an individual mouse. Statistical significance was determined via ordinary two-way ANOVA with Tukey’s multiple comparisons test with a single pooled variance. ****P < 0.0001, ***P = 0.0001–0.001, **P = 0.001–0.01, *P = 0.01–0.05.

In the breakthrough infection study, we measured vaccine-specific total IgG, IgG1, and IgG2a titers similar to analyses done for the OVA-sensitization mice. High titers of TIV-specific total IgG were detected in the two vaccinated groups at 2 weeks post-vaccination (Fig. 7A). TIV-specific total IgG titers continued to rise post-challenge for both TIV-vaccinated groups, although NC99-challenged mice had significantly higher titers than the mock-challenged group at 10 DPC, suggesting a systemic boosting effect from encountering viral antigen in the respiratory tract. TIV-specific IgG titers remained undetectable in PBS-vaccinated mice until 10 DPC following influenza infection, indicative of a *de novo* immune response against NC99. PBS-vaccinated NC99-challenged primary infection mice had higher TIV-specific titers by 28 DPC but still had significantly lower titers compared to mice that were TIV-vaccinated. Similar patterns were observed for both IgG1 and IgG2a, although both isotypes were not as detectable for the primary infection mice at 10 DPC (Fig. 7B and C). Additionally, TIV-specific IgG2a titers were significantly higher in TIV-vaccinated NC99-challenged breakthrough infection mice compared to TIV-vaccinated mock-challenged controls, but this difference was not statistically significant for TIV-specific IgG1 titers. Given the similar increases in both IgG1 and IgG2a titers following vaccination and challenge, all TIV-vaccinated mice exhibited balanced IgG2a/IgG1 ratios through 3 DPC (Fig. 7D). At 7 and 10 DPC, the breakthrough infection mice had more skewing towards IgG2a relative to mock-challenged controls, although this skewing was transient, and the ratios resembled the mock-challenged controls by 28 DPC. The PBS-vaccinated NC99-challenged mice undergoing a primary influenza infection, dogmatically Type 1-biased event, had a significantly IgG2a-skewed response that was evident at 28 DPC. There were transiently higher concentrations of total serum IgE in breakthrough infection mice at 3 DPC, although not as high as measurements in the OVA-sensitized, allergic mice (Fig. 7E).

Overall, our analyses of the antigen-specific response in the serum were in line with lung immune cell quantification, cytokine and chemokine measurement, and histopathology. The overt Type 2 bias observed in OVA-sensitized mice was corroborated by high OVA-specific IgG1 titers with little to no IgG2a. Similarly, the Type 1 biased lung response in primary influenza infection mice was mirrored in the IgG2a-skewed serum responses to antigen at later time points post-challenge. Breakthrough infection mice had balanced IgG2a/IgG1 ratios, indicative of a balanced Type 1/2 response.

### PCA of multiple host immune metrics shows a distinct, balanced Type 1/2 response for breakthrough infection mice

**Fig 8:**
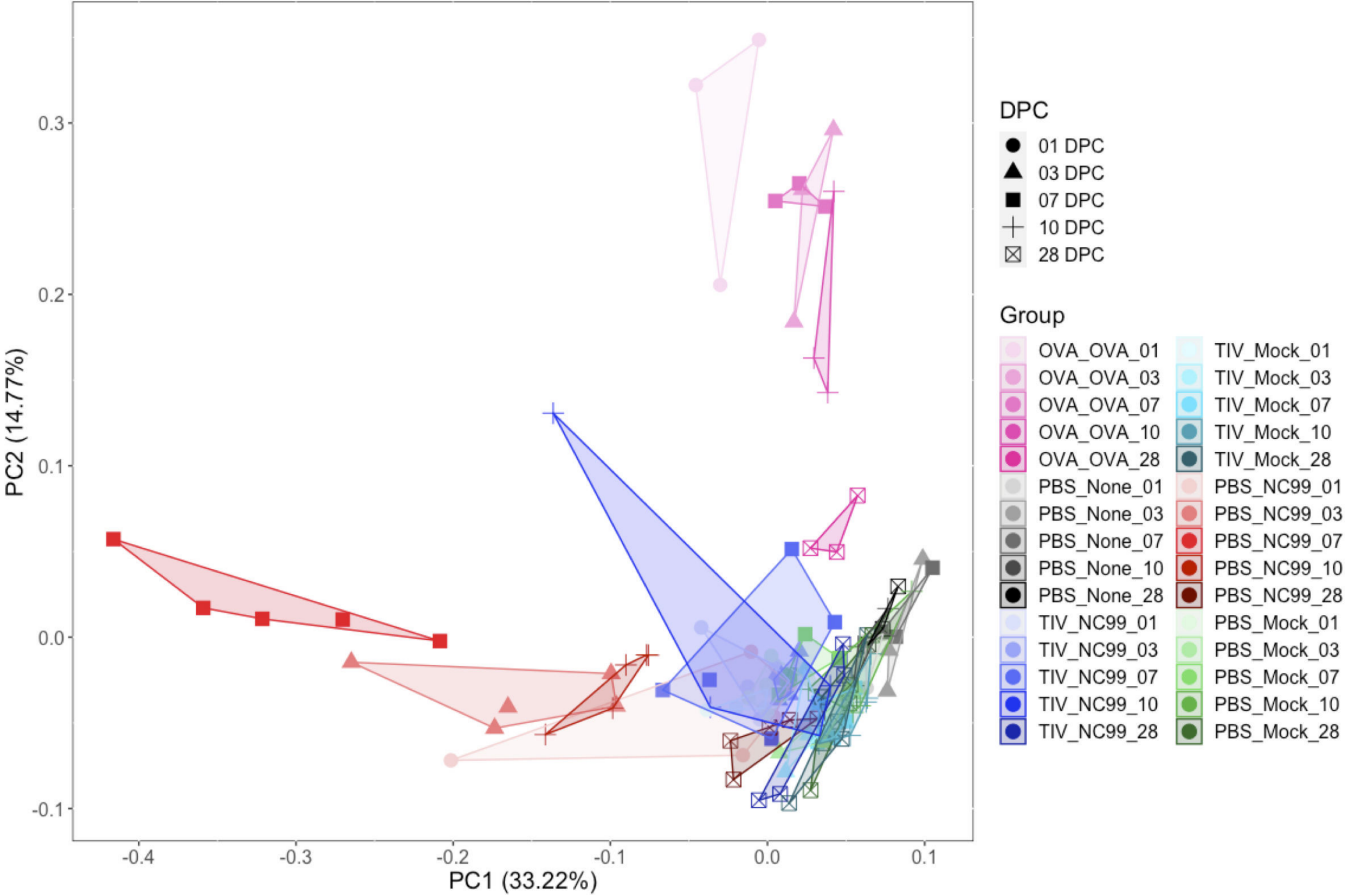
PCA confirms balanced systemic host immune response in breakthrough infection mice relative to primary infection and OVA mice. A total of 67 different immune metrics from each mouse were used for PCA: the counts of 5 immune cell populations, body weight percentages from up to 12 time points, concentrations of 27 cytokines/chemokines, pathology scores for 19 different metrics, goblet cell scores, and total IgE concentration from three time points. Antigen-specific IgG, IgG1, IgG2a, IgG2a/IgG1 ratios, and TCID_50_ values were excluded from analysis to generate antigen-agnostic immune profiles. Symbol colors denote treatments, and symbol shapes denote time point post-challenge. Each symbol represents an individual mouse.

Given the large number of individual-level data collected for each mouse in these studies, we used PCA to visualize differences between treatment groups and time points (Fig. 8). We integrated a total of 67 parameters: immune cell counts from flow cytometry, body weight percentages, cytokine and chemokine concentrations, histopathology scores, mucin staining scores, and total IgE concentrations. Antigen-specific antibody measurements or influenza-specific assay results were omitted to allow for head-to-head comparison of all treatment conditions at once. Distinct profiles for primary infection, breakthrough infection, and OVA sensitization were observed. Differences in immune profile by time point could also be observed: the primary infection mice at 7 DPC and OVA-sensitized mice at 1 DPC varied the most along PC1 and PC2, respectively, compared to mock-challenged controls. Additionally, later time points, such as 28 DPC, for both conditions clustered more closely to mock-challenged controls. Primary infection in mice varied the most on principal component 1 (PC1), driven by metrics such as body weight loss, AM number, and IL-33 concentration (Fig. S8). OVA-sensitized mice varied the most on principal component 2 (PC2), driven by eosinophil counts via flow cytometry and pathology, and concentrations of canonical Type 2 cytokines such as IL-4 and IL-5. Breakthrough infection mice had an intermediate immune profile between the primary infection and OVA-sensitized mice, clustering close to mock-challenged controls.

### Imaging confirms cellular dynamics seen by flow cytometry, identifies cell-cell interactions in the lung space, and visualizes granulocyte extracellular trap formation

**Fig 9:**
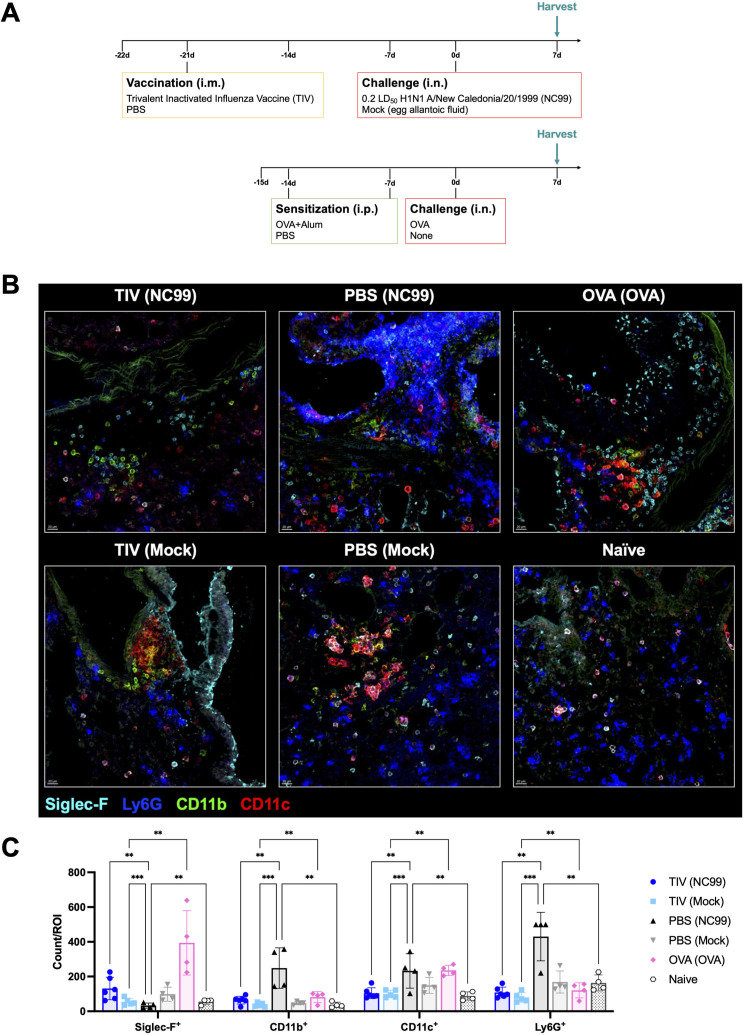
Multiparameter confocal microscopy confirms immune cell dynamics observed by flow cytometry in the lung at 7 DPC. Lung sections were stained for Siglec-F, CD11b, CD11c, and Ly6G to visualize major myeloid populations. (**A**) Study design, (**B**) representative images, and (**C**) number of marker-positive cells were quantified for each region of interest (ROI). Bar plots and error bars represent mean ± SD, with each symbol representing an individual mouse. Scale bar in the bottom left corner of each image represents 20 µm. Statistical significance was determined via ordinary two-way ANOVA with main effects only, and with Tukey’s multiple comparisons test using a single pooled variance. ***P = 0.0001–0.001, **P = 0.001–0.01.

We used multiplex fluorescence imaging to visualize our immune cell populations of interest and understand cell-cell interactions for all models used (Fig. 9A). For this study, we included all four breakthrough infection model groups, as well as an OVA-sensitized group as a positive control for lung eosinophilia and a naive, negative control group as a baseline. Based on data from our kinetics studies, we moved forward with 7 DPC as our time point of interest since our primary focus is in lung eosinophils during breakthrough infection, with a special emphasis on Siglec-F^hi^ eosinophils. Although total eosinophil numbers were the highest at 10 DPC, we selected 7 DPC due to more substantial enrichment for the Siglec-F^hi^ subset at this time point.

First, we quantified the number of Siglec-F^+^ (eosinophils and AMs), CD11b^+^ (macrophages), CD11c^+^ (dendritic cells), and Ly6G^+^ (neutrophils) cells using microscopy (Fig. 9B and C). In line with findings from flow cytometry, we observed a significantly higher number of Siglec-F^+^ cells per region of interest (ROI) in breakthrough infection mice and OVA-sensitized mice compared to primary infection and TIV (mock), respectively (Fig. 9C). As seen in flow cytometry, the PBS-vaccinated NC99-challenged primary influenza infection mice had significantly higher counts of Ly6G^+^ cells compared to other groups. The primary influenza infection group also had higher counts per ROI of CD11b^+^ cells and CD11c^+^ cells.

**Fig 10:**
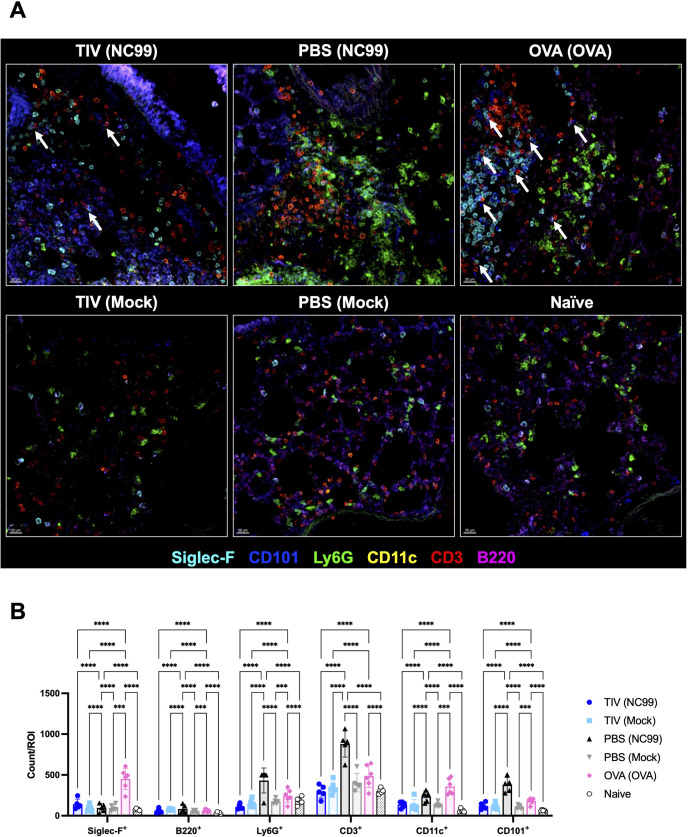
Granulocyte-T-cell interactions can be visualized in the lung parenchymal space following breakthrough infection, primary infection, or allergic sensitization. Lung sections were stained for Siglec-F, CD101, Ly6G, CD11c, CD3, and B220 to quantify cell-cell interactions in tissue. (**A**) Representative images and (**B**) number of marker-positive cells were quantified for each ROI. White arrows denote regions of CD101^+^ Siglec-F^+^ cells interacting with CD3^+^ cells. Bar plots and error bars represent mean ± SD, with each symbol representing an individual mouse. The scale bar in the bottom left corner of each image represents 20 µm. Statistical significance was determined via ordinary two-way ANOVA with main effects only, and with Tukey’s multiple comparisons test using a single pooled variance. ****P < 0.0001, ***P = 0.0001–0.001.

Next, we stained lung sections for Siglec-F (eosinophils and AMs), Ly6G (neutrophils), CD11c (dendritic cells), CD3 (T cells), and B220 (B cells) to visualize granulocyte-lymphocyte interactions in the lung (Fig. 10A). We also included CD101 to identify inflammatory, activated eosinophils. Building upon data from the previous imaging panel and our flow cytometry data, we also observed a significantly higher number of CD3^+^ and CD101^+^ cells in the lungs of PBS-vaccinated NC99-challenged primary infection mice (Fig. 10B). We observed a multitude of interactions between Siglec-F^+^ cells and Ly6G^+^ cells with CD3^+^ cells (Fig. 10A). Of note, we noticed multiple areas of CD101^+^ Siglec-F^+^ cells interacting with CD3^+^ cells in the OVA-sensitized lungs, potentially indicating activated eosinophils interfacing with T cells (Fig. S9).

**Fig 11:**
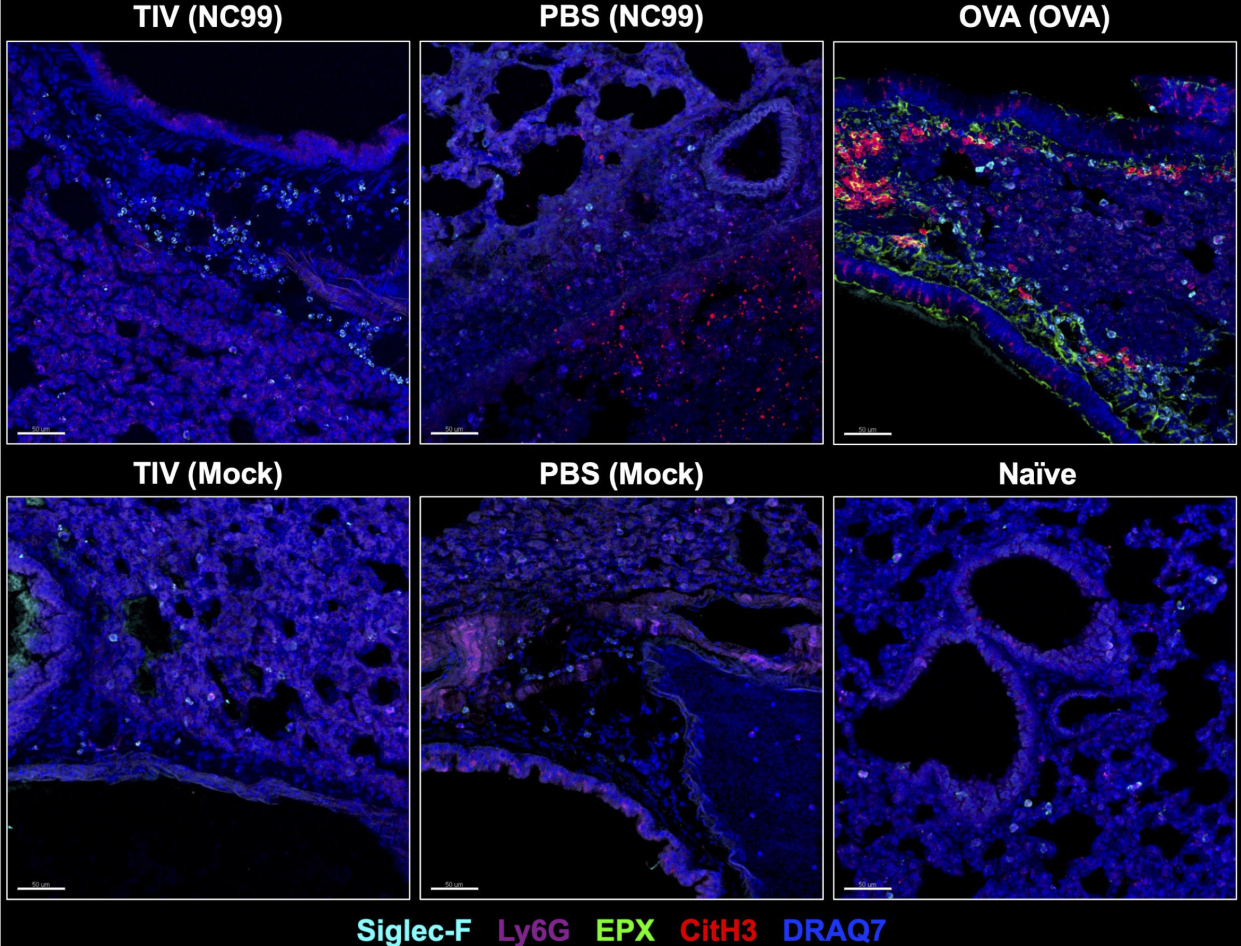
Extracellular trap release is most prominently seen after allergic sensitization or primary infection, but not breakthrough infection. Lung sections were stained with Siglec-F, Ly6G, EPX, and citrullinated H3 antibodies alongside a DNA stain, DRAQ7, to evaluate neutrophil (CitH3^+^) and eosinophil extracellular trap (cell-free EPX^+^) formation in tissue. Scale bar in the bottom left corner of each image represents 50 µm. Representative images of each treatment group are shown here.

Since neutrophil or eosinophil extracellular traps (NETs and EETs, respectively) can exacerbate host lung disease during respiratory viral infection or asthma, we stained lung sections to check for the presence of NETs or EETs (Fig. 11). NETs have some properties that aid in clearing viral infection, such as direct antiviral activity against influenza, conferred by arginine-rich histones H3 and H4 released during NET formation (50, 51). However, NETs have also been shown to damage lung tissue, promote inflammation, and decrease gas exchange, thereby significantly increasing disease severity (51–53). EETs have been predominantly described in the context of extracellular pathogens, such as bacteria, and implicated in the pathogenesis of asthma (54–56). We also stained sections for Siglec-F^+^ cells and Ly6G^+^ cells, as well as a DNA stain to identify nuclei (DRAQ7^+^). Qualitatively, we observed Siglec-F^+^ cells in the lungs of TIV-vaccinated NC99-challenged breakthrough infection mice, but in the absence of cell-free eosinophil peroxidase (EPX). This is in stark contrast with the OVA-sensitized mice, which had multiple areas with extensive cell-free EPX and citrullinated H3 staining, indicative of EET and NET formation, respectively (57, 58). NET formation was also prominently observed in PBS-vaccinated NC99-challenged primary influenza infection mice. NET formation in the lungs following viral challenge has been described for primary SARS-CoV-2 infection (59).

## DISCUSSION

This study provides a comprehensive, longitudinal analysis of immune responses in the lungs across three distinct conditions: OVA sensitization as a typical Type 2 response, primary influenza infection as a Type 1 response, and breakthrough influenza infection in vaccinated hosts as a mixed Type 1/2 response. By examining the kinetics of immune cell populations, cytokine and chemokine profiles, and lung pathology, critical differences in the dynamics and consequences of these responses were revealed. Using imaging to evaluate differences in treatment groups at 7 DPC, a time point exhibiting high eosinophils in OVA-sensitized and breakthrough infection mice, we visualized granulocyte-T-cell interactions in the lung tissue space alongside NET and EET formation.

OVA sensitization induced a classic Type 2 immune response characterized by robust eosinophilic infiltration, transient neutrophilia, and AM depletion during the acute phase. Eosinophil recruitment in OVA-sensitized mice was particularly dramatic, with a marked enrichment of the Siglec-F^hi^ subset, which is thought to be an activated, pro-inflammatory subset (25, 43–45). This response was driven by elevated Type 2 cytokines, including IL-4, IL-5, and IL-13, which are known to promote goblet cell hyperplasia and mucin production, hallmark features of allergic inflammation (41, 60). Findings in the lungs were corroborated by analysis of the antigen-specific humoral response: OVA-sensitized mice generated an IgG1-dominated OVA-specific response, which is indicative of Th2-skewing, and high concentrations of total IgE in the serum (47–49).

In contrast, primary influenza infection elicited a strong Type 1 response dominated by neutrophilic inflammation and prolonged AM depletion. The cytokine profile was consistent with antiviral immunity, with high levels of IFN-γ, TNF-α, and IL-12. Unlike the OVA sensitization model, there was no substantial eosinophilia post-challenge, and significant lung pathology was observed. Viral load measurements indicated prolonged viral persistence in primary infection mice compared to vaccinated breakthrough infection mice, likely due to the lack of pre-existing immunity. Characterization of the humoral response post-challenge confirmed elicitation of a Th1-skewed response, with IgG2a being the dominant isotype (47–49).

Breakthrough influenza infection in vaccinated mice presented a mixed Type 1/2 response, without the heavy Type 1 skewing observed in primary infection mice. Eosinophil recruitment was moderate but significant, with an enrichment of the Siglec-F^hi^ subset particularly at 7–10 DPC, although not to the extent observed in OVA-sensitized mice. Importantly, AM counts were preserved, and neutrophilic inflammation was mild, suggesting that vaccination mitigates excessive immune activation. Cytokine and chemokine levels in breakthrough infection mice were balanced, with muted Type 1 and Type 2 modules compared to primary infection and OVA sensitization, respectively. Surprisingly, concentrations of canonical Type 2 cytokines IL-4, IL-5, and IL-13 were highest in the primary infection mice, despite the lack of eosinophilia (Fig. S5). Overall, the balanced immune response in breakthrough infection mice coincided with rapid viral clearance and minimal lung disease, emphasizing the protective effects of pre-existing immunity conferred by vaccination.

Vaccination significantly altered the immune landscape in breakthrough infection, preserving AM populations and preventing the severe neutrophilic inflammation observed in primary influenza infection. AMs are essential for maintaining lung homeostasis and orchestrating immune responses, and their preservation likely contributed to the regulated inflammatory environment in vaccinated hosts. Moreover, it is to be expected that preservation of AMs could have allowed for faster recovery and clearance of damaged cells, a metric for a more rapid return to homeostasis. Additionally, the absence of NETs and EETs in breakthrough infection mice underscores the protective nature of vaccine-induced immunity. Extracellular traps are associated with tissue damage and can exacerbate disease in severe viral infections and allergic diseases, to the detriment of the host (54–56). Their absence in the vaccinated hosts highlights the efficacy of vaccination in preventing excessive inflammation. The rapid viral clearance observed in vaccinated mice underscores the benefits of pre-existing immunity. Reduced viral titers correlated with minimal weight loss and quicker recovery, emphasizing the ability of vaccination to mitigate disease severity. These findings align with clinical observations of reduced morbidity in vaccinated individuals experiencing breakthrough infections, providing strong evidence for the effectiveness of influenza vaccines.

These findings reinforce the distinction between eosinophilic recruitment in mouse models of breakthrough influenza infection and VAERD. VAERD is characterized by severe disease, including goblet cell hyperplasia and excessive Type 2 cytokine responses, none of which were observed in breakthrough infection. While eosinophilia was present in vaccinated mice, it was non-immunopathological and associated with reduced viral titers and minimal lung damage. This suggests that rather than exacerbating disease, eosinophils may contribute to host defense or tissue repair when excessive inflammation is not present in the local lung environment. The enrichment of Siglec-F^hi^ eosinophils, often implicated in allergic conditions, did not reach the pathological levels observed in OVA-sensitized mice. In line with the Local Immunity And/or Remodeling/Repair (LIAR) hypothesis (14), we speculate that the Siglec-F^hi^ eosinophils observed during breakthrough infection may be functionally distinct from the Siglec-F^hi^ eosinophils observed during allergic inflammation, due to differences in the host lung microenvironment; OVA-sensitized mice were extremely polarized toward Type 2 responses, whereas breakthrough infection mice have a more balanced Type 1/2 response (Fig. 8). Whether or not the wealth of Type 2 cytokines, chemokines, and other factors confer substantial functional differences to eosinophils in the lungs of allergen-challenged mice versus breakthrough infection mice remains to be seen, particularly for the Siglec-F^hi^ subset. In-depth phenotyping or transcriptomic analysis of eosinophils across multiple different inflammatory states will shed light on the differences between eosinophil subsets. Additionally, it is also possible that peak mucin staining and regenerative activity in the lungs could be occurring at later timepoints, such as 14 or 21 DPC, in the breakthrough and primary infection mice. As such, we hope to investigate these later timepoints in future studies.

The specific role of eosinophils in breakthrough respiratory infection remains an area of interest. Traditionally associated with allergic inflammation, eosinophils have emerging roles in antiviral defense, such as antigen presentation and cytokine production (9–13). In this study, eosinophilia in vaccinated mice was correlated with rapid viral clearance and reduced inflammation, suggesting a protective function. We also observed Siglec-F^+^ cells interacting with CD3^+^ T cells, most prevalently in OVA-sensitized mice (Fig. 10; Fig. S9). Wiese et al. have also observed inflammatory eosinophils (Siglec-F^+^CCR3^+^CD11c^+^) interacting with T cells in the lungs of mice with allergic asthma, which were able to promote antigen-specific proliferation and differentiation of naive T cells (61). We also visualized activated, inflammatory eosinophils (CD101^+^ Siglec-F^+^ cells) in contact with T cells in the lung. Although the number of interactions was not statistically significant, the specific nature of the eosinophil-CD3 cell interactions may have a role in immune responses in the lung, such as during breakthrough infection. It is possible that eosinophils were presenting antigen to T cells, although it is unlikely that there were high levels of viral antigen present at the time point selected for imaging; by 7 DPC, viral titers are entirely controlled in breakthrough infection mice. Other possible eosinophil-T-cell interactions of interest include immunomodulation (12, 62). CD101 is associated with highly immunosuppressive Tregs and myeloid cells (12, 63–65). Whether or not CD101^+^ eosinophils are able to suppress T cells has not been reported, but it is a possible function. Other putative roles for eosinophils during breakthrough infection of respiratory viruses in vaccinated hosts have been reviewed by Chang and Schotsaert (12). However, the definitive roles of eosinophils during breakthrough infection or why they are recruited have yet to be defined. Future research should investigate the specific mechanisms by which eosinophils contribute to immune defense in this context and delineate the thresholds at which eosinophilia transitions from protective to pathological. We are currently investigating the precise contribution of eosinophils during breakthrough infection by leveraging eosinophil-specific depletion models or eosinophil-depleting antibodies, in conjunction with functional studies and single-cell profiling.

One of the goals of our longitudinal study was to identify a recruitment signal for eosinophils during breakthrough infection via cytokine and chemokine profiling. We did not find a clear, conclusive recruitment signal for eosinophils in the breakthrough infection model. In general, most cytokine or chemokine expression patterns we observed in breakthrough infection mice were similar to those of primary infection mice, except substantially lower in concentration. We suspect this is because the strength of the cytokine/chemokine signal is directly linked to the viral titer; primary infection mice had significantly higher viral load while breakthrough infection mice only had detectable viral load through 3 DPC before rapidly controlling viral replication (Fig. 3C). Of the 27 cytokines and chemokines we evaluated, three analytes deviated from the viral titer-associated rise-fall kinetics: CCL11, IL-27, and IL-33. CCL11, also known as Eotaxin-1, is a potent eosinophil chemoattractant (66). Interestingly, CCL11 measurements proved inconclusive in the OVA-sensitization model, as OVA-sensitized mice did not have significantly higher concentrations than PBS control mice (Fig. S4). Similarly, CCL11 concentrations were highest in the breakthrough infection mice only at 1 DPC, much earlier than peak lung eosinophilia at 7-10 DPC (Fig. S5). At other time points, CCL11 concentrations in breakthrough mice were not significantly higher than any of the other groups. We expected to see classic rise-fall kinetics in the concentrations of CCL11 and IL-5 for the breakthrough infection mice, corresponding with the gradual influx of lung eosinophil counts, but the kinetics for these two analytes did not appear clearly linked to eosinophil numbers. It is possible that the chemokines or cytokines were already bound by cells and not detectable as a measurable, free-floating protein in the lung homogenate supernatants. Next, IL-27 also had unique expression patterns across the different treatment groups we evaluated. IL-27 is an immunomodulatory cytokine produced by antigen-presenting cells in response to toll-like receptor (TLR) stimulation that acts on both innate and adaptive cells (67). It has been linked to attenuation of allergic airway diseases, such as by repressing group 2 innate lymphoid cells (ILC2) expansion, and has multiple antiviral roles as well (68, 69). During influenza infection, IL-27 can promote transcription of interferon-stimulated genes like *Mx1*, increase the number of influenza-specific IFN-γ+ CD8 T cells, and ameliorate immunopathology by stimulating T cell production of IL-10 (69). IL-27 concentrations were the most elevated in OVA-sensitized mice at 1 DPC and in primary infection mice at 7 DPC, time points that corresponded to peak inflammation and pathology (Fig. 4; Fig. S5). It is likely that IL-27 expression increased at these specific time points as a negative feedback mechanism to dampen inflammation. Of note, IL-27 concentrations were the lowest in the breakthrough infection mice at 3 DPC compared to all other groups, including mock-challenged controls, despite detectable viral titers at this time point (Fig. 3C; Fig. S5). We speculate that below a certain inflammatory threshold, IL-27 expression is actively suppressed: because the sublethal infection was well-controlled by factors such as pre-existing vaccine-elicited antibodies in the breakthrough infection mice, the magnitude of inflammation triggered was lower, resulting in less immunopathology and therefore less need to induce a marked anti-inflammatory host state. In line with this idea, BALT hyperplasia was more prevalent in primary infection mice, but not breakthrough infection mice; iBALT formation is correlated with tissue damage (Fig. S7B) (70). Altogether, the absence of overt tissue damage in vaccinated hosts experiencing breakthrough infection, relative to unvaccinated hosts, resulted in muted pro- and anti-inflammatory signatures. Lastly, IL-33 was uniquely reduced in the primary infection mice (Fig. S5). IL-33 is an alarmin cytokine released in response to tissue damage that can potently activate Type 2 immune effector cells such as ILC2s (71). Our findings contrast with a previous report that primary influenza infection of C57BL/6 mice increases the number of IL-33 mRNA transcripts in the lungs; we observed a significant reduction of IL-33 protein, potentially highlighting mouse strain-specific differences in lung immunity since we worked with BALB/c mice (72). Of note, the kinetics of IL-33 concentrations in primary infected mice paralleled that of AM counts, gradually decreasing through 7–10 DPC to significantly lower levels compared to other groups. No such reduction in IL-33 or AMs was observed in breakthrough infection mice, which had vaccine protection from sustained viral replication. Whether or not IL-33 and AM survival are linked has yet to be described, although IL-33 has been shown to activate AMs in a mouse model of asthma (73). Collectively, although we did not identify a clear cytokine or chemokine recruitment signal for eosinophils into the lungs that neatly coincided with the timing of peak eosinophilia, it is possible that other factors, like complement or lipid mediators, are responsible for recruiting eosinophils in this scenario. Eosinophils can also be recruited via the C5a/C5aR1 axis (61), serotonin metabolite 5-hydroxyindoleacetic acid (5-HIAA) binding to cell surface GPR35 (74), and arachidonic acid metabolites (75). Eotaxin-2 can also recruit eosinophils and was not within the cytokine/chemokine detection panel we used for these experiments, but will be included in future studies (75).

Our study is believed to be the first to longitudinally profile breakthrough influenza infection across multiple time points and directly compare it to primary influenza infection and allergic sensitization in the lungs. Our detailed kinetic comparisons integrated flow cytometry, cytokine and chemokine analysis, morbidity, serology, imaging, and histopathology, providing a comprehensive, holistic view of immune dynamics. The high number of different immune metrics collected on an individual mouse basis allowed us to identify distinct immune profiles using unbiased methods. We also visualized novel CD101^+^Siglec-F^+^ cell and CD3^+^ T-cell interactions, which we aim to investigate further in future studies. Additionally, we demonstrate that imaging of granulocyte extracellular traps can provide insight into host immunity and lung health: staining for both EET and NET formation in lung sections can be used to visually determine pathological granulocyte function.

Although our study has multiple strengths, certain limitations of the study warrant consideration. The lack of perfusion or intravascular staining before lung harvest may have influenced immune cell counts, as some of the numbers could reflect circulating cells rather than true tissue infiltration. However, CD101^+^ Siglec-F^hi^ eosinophils have been demonstrated to be in the lung tissue by multiple independent groups, as this population is predominantly protected from intravascular staining (43, 61, 76, 77). Furthermore, we confirmed the enhanced presence of Siglec-F^+^ cells in the lung tissue via microscopy. Therefore, we are confident that our quantification of Siglec-F^hi^ eosinophils reflects a lung-specific population, rather than a circulating population. Given the scope of the study, we did not include a post-prime pre-challenge lung harvest. The absence of a pre-challenge time point limits the ability to assess whether vaccination itself remodeled the lung immune environment. The effects of vaccination on tissues distal from the injection site, such as training and alterations of bone marrow progenitor cell populations, will be evaluated in future studies. Ultimately, we present a mouse model for breakthrough influenza infection in vaccinated hosts; confirmation that eosinophils are recruited to the lungs of humans experiencing breakthrough infection is warranted, perhaps through the collection of bronchoalveolar lavage fluid or sputum. Continued surveillance of patients on eosinophil-depleting treatments and the effect of these treatments on their vaccine immune responses, particularly the incidence rate and severity of breakthrough infections in eosinophil-depleted patients, would be of great interest as well.

In conclusion, this study demonstrates that breakthrough influenza infection in vaccinated hosts elicits a balanced Type 1/2 immune response characterized by moderate eosinophilia, preserved AM counts, and minimal pathology. Unlike RSV VAERD, eosinophilia in breakthrough infection does not appear to be a cause for concern or a side effect of off-target vaccine immunity; this instance of lung eosinophilia was non-pathological and corresponded to enhanced immune defense. These findings underscore the protective role of vaccination in modulating immune responses, preventing excessive inflammation, and promoting rapid recovery. Our findings also highlight the potential for eosinophils to contribute positively to antiviral immunity under specific, balanced conditions. Our work has significant implications for understanding vaccine-induced immunity and optimizing vaccine strategies to achieve effective and safe protection against respiratory viral infections without enhancing immune pathology by clarifying that the induction of eosinophils is not always deleterious and may not always warrant alarm.

## Data Availability

The raw data supporting the conclusions of this article will be made available by the authors, without undue reservation.

## SUPPLEMENTAL MATERIAL

Figure S1 jvi.00965-25-s0001.tiff Gating strategy for flow cytometry of the lungs.

Figure S2 jvi.00965-25-s0002.tiff The majority of Siglec-F^hi^ eosinophils express CD101.

Figure S3 jvi.00965-25-s0003.tiff Significant enrichment for the Siglec-F^hi^ subset of eosinophils occurs only in OVA sensitized mice or breakthrough infection mice.

Figure S4 jvi.00965-25-s0004.tiff Cytokine and chemokine concentrations in lung homogenate supernatants from OVA-sensitized mice and controls.

Figure S5 jvi.00965-25-s0005.tiff Cytokine and chemokine concentrations in lung homogenate supernatants from breakthrough infection mice and controls.

Figure S6 jvi.00965-25-s0006.tiff Pathology scores for OVA-sensitized mice and controls.

Figure S7 jvi.00965-25-s0007.tiff Pathology scores for breakthrough infection mice and controls.

Figure S8 jvi.00965-25-s0008.tiff PCA loadings.

Figure S9 jvi.00965-25-s0009.tiff CD101^+^Siglec-F^+^ and CD3^+^ cell-cell interactions quantified per region of interest.

Supplemental legends jvi.00965-25-s0010.docx Legends for Fig. S1 to S9.

ASM does not own the copyrights to Supplemental Material that may be linked to, or accessed through, an article. The authors have granted ASM a non-exclusive, world-wide license to publish the Supplemental Material files. Please contact the corresponding author directly for reuse.
