# Supplementary material for: Transient lung eosinophilia during breakthrough influenza infection in vaccinated mice is associated with protective and balanced Type 1/2 immune responses
Source: J Virol. 2025 Nov 5;99(11):e00965-25. doi: 10.1128/jvi.00965-25 (PMC12646012; doi:10.1128/jvi.00965-25)
Supplement: Supplemental legends — Legends for Fig. S1 to S9. [file jvi.00965-25-s0010.docx]

**Supplementary Figures**

**Figure S1.** Gating strategy for flow cytometry of the lungs. Representative mouse from the breakthrough infection group at 7 DPC is shown here.

**Figure S2. The majority of Siglec-F^hi^ eosinophils express CD101.** Frequency of CD101^+^ cells within the Siglec-F^hi^ eosinophil population was measured for (**A**) OVA sensitized mice or (**B**) breakthrough infection mice and controls. Statistical significance was determined via ordinary two-way ANOVA (**A**) with Šídák’s multiple comparisons test with a single pooled variance or (**B**) with Tukey’s multiple comparisons test with a single pooled variance. **P = 0.001 to 0.01, *P = 0.01 to 0.05.

**Figure S3. Significant enrichment for the Siglec-F^hi^ subset of eosinophils only occurs in OVA sensitized mice or breakthrough infection mice.** Frequency of Siglec-F^hi^ eosinophils within the total eosinophil population in (**A**) OVA-sensitized mice and controls or (**B**) breakthrough infection mice and controls. Frequency of Siglec-F^hi^ eosinophils within the total eosinophil population in (**C**) OVA-sensitized mice and controls or (**D**) breakthrough infection mice and controls. Statistical significance was determined via ordinary two-way ANOVA (**A**, **C**) with Šídák’s multiple comparisons test with a single pooled variance or (**B, D**) with Tukey’s multiple comparisons test with a single pooled variance. ****P < 0.0001, ***P = 0.0001 to 0.001, **P = 0.001 to 0.01, *P = 0.01 to 0.05.

**Figure S4. Cytokine and chemokine concentrations in lung homogenate supernatants from OVA-sensitized mice and controls.** Statistical significance was determined via ordinary two-way ANOVA with Šídák’s multiple comparisons test with a single pooled variance. ****P < 0.0001, ***P = 0.0001 to 0.001, **P = 0.001 to 0.01, *P = 0.01 to 0.05.

**Figure S5. Cytokine and chemokine concentrations in lung homogenate supernatants from breakthrough infection mice and controls.** Statistical significance was determined via ordinary two-way ANOVA with Tukey’s multiple comparisons test with a single pooled variance. ****P < 0.0001, ***P = 0.0001 to 0.001, **P = 0.001 to 0.01, *P = 0.01 to 0.05.

**Figure S6. Pathology scores for OVA-sensitized mice and controls.** Scores for metrics for the (**A**) total lung; (**B**) bronchi and bronchioles, peribronchial and peribronchiolar regions; (**C**) alveoli and alveolar septa. Statistical significance was determined via ordinary two-way ANOVA with Šídák’s multiple comparisons test with a single pooled variance. ****P < 0.0001, ***P = 0.0001 to 0.001, **P = 0.001 to 0.01, *P = 0.01 to 0.05.

**Figure S7. Pathology scores for breakthrough infection mice and controls.** Scores for metrics for the (**A**) total lung; (**B**) bronchi and bronchioles, peribronchial and peribronchiolar regions; (**C**) alveoli and alveolar septa. Statistical significance was determined via ordinary two-way ANOVA with Tukey’s multiple comparisons test with a single pooled variance. ****P < 0.0001, ***P = 0.0001 to 0.001, **P = 0.001 to 0.01, *P = 0.01 to 0.05.

**Figure S8. PCA loadings.** Bar plot of loadings for (**A**) PC1 and (**B**) PC2.

**Figure S9. CD101^+^Siglec-F^+^ and CD3^+^ cell-cell interactions quantified per region of interest.** Statistical significance was determined using a Kruskal-Wallis test with Dunn’s multiple comparisons test. **P = 0.001 to 0.01.
